# *S. pombe* Kinesins-8 Promote Both Nucleation and Catastrophe of Microtubules

**DOI:** 10.1371/journal.pone.0030738

**Published:** 2012-02-20

**Authors:** Muriel Erent, Douglas R. Drummond, Robert A. Cross

**Affiliations:** Centre for Mechanochemical Cell Biology, Warwick Medical School, The University of Warwick, Coventry, United Kingdom; University of Minnesota, United States of America

## Abstract

The kinesins-8 were originally thought to be microtubule depolymerases, but are now emerging as more versatile catalysts of microtubule dynamics. We show here that *S. pombe* Klp5-436 and Klp6-440 are non-processive plus-end-directed motors whose in vitro velocities on *S. pombe* microtubules at 7 and 23 nm s^−1^ are too slow to keep pace with the growing tips of dynamic interphase microtubules in living *S. pombe*. In vitro, Klp5 and 6 dimers exhibit a hitherto-undescribed combination of strong enhancement of microtubule nucleation with no effect on growth rate or catastrophe frequency. By contrast in vivo, both Klp5 and Klp6 promote microtubule catastrophe at cell ends whilst Klp6 also increases the number of interphase microtubule arrays (IMAs). Our data support a model in which Klp5/6 bind tightly to free tubulin heterodimers, strongly promoting the nucleation of new microtubules, and then continue to land as a tubulin-motor complex on the tips of growing microtubules, with the motors then dissociating after a few seconds residence on the lattice. In vivo, we predict that only at cell ends, when growing microtubule tips become lodged and their growth slows down, will Klp5/6 motor activity succeed in tracking growing microtubule tips. This mechanism would allow Klp5/6 to detect the arrival of microtubule tips at cells ends and to amplify the intrinsic tendency for microtubules to catastrophise in compression at cell ends. Our evidence identifies Klp5 and 6 as spatial regulators of microtubule dynamics that enhance both microtubule nucleation at the cell centre and microtubule catastrophe at the cell ends.

## Introduction

Microtubule dynamics allow cells to rapidly assemble, remodel, or disassemble polarized arrays of microtubules. Pure tubulin in vitro shows intrinsic dynamic instability, whereby microtubules spontaneously nucleate, grow steadily, and then spontaneously and rapidly depolymerise [1]. Dynamic instability is well described by four parameters: growth rate, shrinkage rate, catastrophe frequency (how often microtubules switch from growth to shrinkage) and rescue frequency (how often they switch from shrinkage to growth) [2]. In cells, these parameters are all heavily regulated [3].

Amongst the regulators are members of the kinesin-13, -14 and -8 families, which modulate the catastrophe frequency [4]. MCAK and related kinesins-13 can diffuse along the microtubule lattice to the growing microtubule tip and drive tubulin subunits to dissociate, either alone or in complex with the MCAK [5], driven by an ATPase cycle that is distinct from that of translocating kinesins [6]. Kar3, a kinesin-14, is a minus end directed translocase that heterodimerises with Cik1, targets the plus ends of taxol-stabilised microtubules, and depolymerizes them at a rate dependent on the taxol concentration [7]. The kinesins-8 are plus end-directed translocases [8], [9], [10], [11], [12], at least one of which, *S. cerevisiae* Kip3, can step processively along the lattice of GMPCPP microtubules to their plus ends, where it enhances the off-rate of GMPCPP tubulin heterodimers [10]. The tail of Kip3 contains a microtubule and tubulin-heterodimer binding site that enhances its processivity and microtubule end binding [13]. Although, like MCAK, Kip3 ATPase is stimulated by tubulin heterodimers [9] the mechanism of depolymerisation is different. Accumulation of Kip3 at MT ends depends on Kip3 translocase activity and requires cooperation between Kip3 molecules, in that Kip3-tubulin complexes are displaced from the microtubule tip by the arrival of another Kip3 [14]. The cell biology of Kip3 is consistent with this type of length-dependent catastrophe mechanism operating in vivo. The velocity of Kip3 in vivo is 47–73 nm s^−1^ [9], [10], similar to its single molecule velocity of 50 nm s^−1^ on brain microtubules in vitro [10], and is sufficient to account for its accumulation at the ends of microtubules growing at 23 nm s^−1^ in vivo [9], [15]. Deletion of *kip3* leads to unusually long spindles [16] [17], longer cytoplasmic microtubules, effects on chromosome congression [18] and a decrease in microtubule catastrophe frequency [9] consistent with microtubule depolymerase activity. However in vivo Kip3 also increases microtubule growth rate, rescue frequency and pause duration whilst decreasing shrinkage rate, suggesting Kip3 has a wide range of effects on dynamic microtubules [9]. The tail of Kip3 is required in vivo for the increase in microtubule rescue frequency and reduction in microtubule shrinkage rate. These effects may result directly from binding of the Kip3 tail to microtubules since in vitro the tail reduces the shrinkage rate of GDP microtubules [13].

The cell biology of other kinesins-8 is also only partially consistent with their having solely a microtubule depolymerase activity. Some observations are consistent with depolymerase activity. RNAi knockdown of Klp67a in *Drosophila* S2 cells produces unusually long spindles [19] as do Klp67a mutants in *Drosophila* embryos [20] [21] and mislocalisation of Klp67a to the cytoplasm of interphase cells causes a shortening of microtubules [22], all consistent with depolymerase activity. However in *Drosophila* primary spermatocytes, although Klp67a destabilizes microtubules during pre-anaphase, it is subsequently required to stabilize the central spindle [23] suggesting Klp67a can have both stabilizing and destabilizing effects. Kif18a, a mammalian kinesin-8, accumulates in an ATP-dependent manner at the plus ends of kinetochore microtubules [11], [24], and affects the dynamics of kinetochore oscillations [11], [24], [25]. Like Kip3, processivity and microtubule end binding by Kif18a depends on a microtubule binding site in the Kif18a tail that is essential for its effects in vivo [26], [27]. Kif18a depletion was originally reported to slow chromosome movement [11], but subsequent studies suggest that chromosome movement actually speeds up [24], [25]. Stumpff and colleagues [20] found that Kif18a increased the frequency of kinetochore directional switching, whereas Jaqaman and colleagues [21] found no such effect but suggested instead that Kif18a may promote depolymerisation specifically on the trailing face of the kinetochore to slow chromosome movement. Over-expression of Kif18a in the interphase cytoplasm, in addition to increasing the catastrophe frequency as expected for a depolymerase, also increases microtubule growth rate, rescue frequency and the duration of pauses whilst slowing shrinkage, overall reducing microtubule dynamicity [28]. Intriguingly, although Kif18a depletion produces long microtubules, inhibition of its motor activity by a drug does not [29], suggesting that long microtubules may not arise from loss of Kif18a function but from loss of its interactions with another protein such as CenpE [30]. Depletion of Kif19, a kinesin-8 present in higher eukaryotes, produces monopolar spindles [31] but not other Kif18a depletion phenotypes [19], [32]. The human kinesin-8 Kif18b destabilises microtubules during mitosis, targeting microtubule ends through a combination of motor activity and an EB1 binding site in its tail [33]. However, Kif18b has no significant direct effect on microtubule dynamics, but rather acts by transporting the kinesin-13 MCAK to microtubule ends and forming a complex with EB1 to enhance its end binding [34].

The fission yeast *S. pombe* has two kinesins-8: Klp5 and Klp6, and no kinesins-13 [35], suggesting it may rely heavily on Klp5 and Klp6 to drive microtubule dynamics. Klp5 and Klp6 are both essential for meiosis [36] but non-essential during interphase and mitosis. Deletion of either Klp5 or Klp6 alone or in combination produces unusually long interphase microtubules [36], [37], suggesting that both Klp5 and Klp6 are depolymerases. In mitosis, Klp5 and Klp6 enter the nucleus separately [38] and then localise to the kinetochores and the spindle midzone [36], [37]. Deletion of either Klp5 or Klp6 produces longer mitotic spindles [39], a delay in establishing the metaphase plate, lagging chromosomes and chromosome mis-segregation [37], [39], [40], [41]. Some of these effects may arise from the role of Klp5 and 6 in chromosome attachment [40]. The non-motor domains of Klp5/6 also form part of a complex important for spindle checkpoint silencing that does not depend on Klp5/6 motor activity [42]. Tischer et al [43] carefully mapped the position of interphase microtubule catastrophes in *S. pombe* and found that they largely occur at cell ends, with a frequency that depends on Klp5/6 activity. They also found that Klp5/6 accumulate at microtubule ends, and that catastrophe frequency increased with increasing microtubule length, consistent with a Kip3-like depolymerase mechanism [43], [44]. However, as well as increasing catastrophe frequency, Klp5/6 in vivo is also reported to increase the rescue frequency and growth rate [38]. Overall, the effects of kinesin-8 on interphase microtubules in budding yeast [9], fission yeast [38] and human cells [28] suggest that different members of the kinesins-8 family can increase not only the catastrophe frequency but also the rescue frequency, growth rate and duration of pauses of dynamic microtubules in vivo [28]. Furthermore, these phenomena can arise through either direct or indirect effects of the kinesins-8.

Resolving the mechanisms responsible for the diverse in vivo properties reported for the kinesins-8 requires in vitro reconstitution experiments to distinguish direct from indirect effects. Perhaps influenced by Kip3 work, most reconstitution experiments so far have focused on depolymerisation of brain microtubules stabilized with taxol or GMPCPP. Kip3 depolymerizes GMPCPP brain microtubules [9], [10], [13], [14] but any effect on dynamic microtubules remains to be demonstrated in vitro. For Kif18a the ability to depolymerise GMPCPP stabilised microtubules in vitro is itself controversial [11] [28]. Kif18a also fails to depolymerise Taxol stabilised microtubules, although in the presence of a non-hydrolysable ATP analogue it does sequester tubulin into ring structures similar to those formed by MCAK, suggesting it shares some of MCAKs functional as well as structural features [45]. In assays on dynamic microtubules Kif18a has no effect on the stability of existing microtubules [28], but does block polymerisation of microtubules by acting as a capping protein [26], [28]. In vitro experiments on full-length Klp5 and 6, expressed as a complex in baculovirus, revealed plus-end-directed sliding of brain microtubules at 39 nm s^−1^, whilst Klp6 motor domain alone drives sliding at 56 nm s^−1^ [12]. Klp5/6 is reported to have no in vitro depolymerase activity, either on GMPCPP or Taxol stabilised brain microtubules or on shrinking GDP brain microtubules [12].

Since the biochemical behaviour of kinesin can be different for microtubules from different species [46] we have examined the effect of Klp5/6 on dynamic *S. pombe* microtubules, both in vitro and in vivo. Our data suggest a new working model for Klp5/6 in vivo. We propose Klp5/6 tubulin complexes initially promote the birth of new microtubules, and thereafter continuously land on the growing microtubule tip, attempting to keep pace with the tip as it grows, but only succeeding at cell ends, where the microtubule tips lodge and their growth slows down in compression. Only then, within the context of the cell end, does Klp5/6 promote microtubule catastrophe. This mechanism, which allows Klp5/6 both to promote nucleation and to amplify an intrinsic tendency for microtubule tips to catastrophise under compression, may have relevance to other kinesins-8.

## Results

### Klp5_436_ and Klp6_440_ are both plus-end-directed microtubule translocases

**Figure 1:**
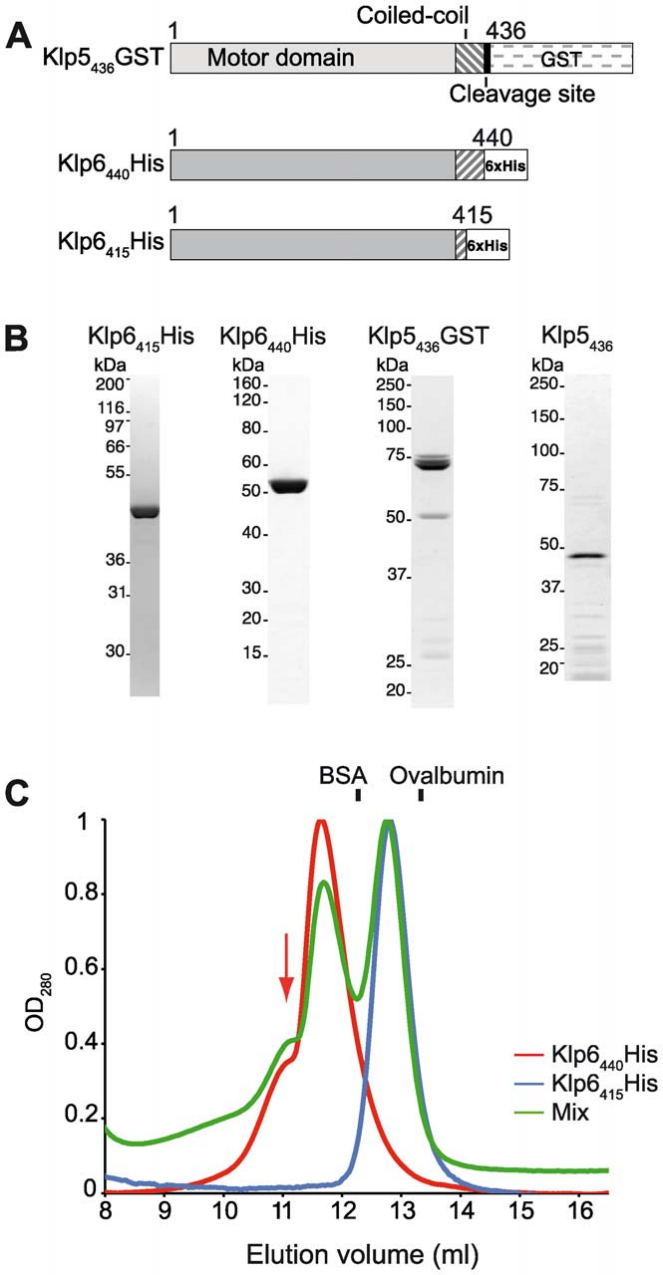
Klp5 and Klp6 constructs and proteins. (**A**) Klp5_436_GST includes the motor domain, the putative coiled-coil domain [36], PreScission protease cleavage site and glutathione-S-transferase (GST) tag. Klp6_440_His contains the motor domain, complete putative coiled-coil domain [36] and 6×His C-terminal tag. Klp6_415_His contains the first 11 amino acids of the coiled coil domain and a 6His C-terminal tag. Predicted molecular weight of constructs are Klp5_436_GST: 74.9 kDa, Klp6_440_His: 50.2 kDa, Klp6_415_His: 47.2 kDa and Klp5_436_ after GST tag cleavage 48.5 kDa. (**B**) Colloidal Coomassie stained SDS-PAGE of desalted Klp6_415_His, microtubule affinity-purified Klp6_440_His; microtubule affinity-purified Klp5_436_GST and Klp5_436_ (Klp5_436_GST after PreScission protease cleavage and purification). (**C**) Klp6_440_His forms a homodimer. Superimposed elution profiles of Superose 12 chromatography of Klp6_415_His (blue), Klp6_440_His (red) and a mixture of Klp6_415_His and Klp6_440_His (green). Elution profiles are consistent with monomeric Klp6_415_His and homodimeric Klp6_440_His. Elution positions of Ovalbumin (43 kDa) and Bovine Serum Albumin (67 kDa) are indicated. Arrowhead indicates the position of a larger complex present in the Klp6_440_His sample.

Since it was unclear if Klp5 is, like Klp6, a microtubule translocase, we expressed Klp5 and 6 alone and in combination. We purified co-expressed full-length Klp5 and Klp6 or full-length Klp6 alone from *E. coli*, but were unable to obtain full length Klp5 alone. Co-expressed proteins were purified by sequential affinity purification using the different tags on Klp5 and 6. After the final affinity purification the ratio of Klp5 to Klp6 present was not 1∶1, suggesting that Klp5 and 6 can form a complex of variable composition (Figure S1). We found evidence that the tail of full length Klp6 mediates an auto inhibition with respect to tubulin heterodimer binding (see below and Table S1), similar to the full-length Klp5/6 complex [12]. In order to address the individual biophysical mechanisms of Klp5 and Klp6 with respect to microtubule dynamics, we focused on two tail-less constructs, Klp5_436_GST and Klp6_440_His. Both constructs contain the motor head domain plus the putative coiled coil dimerisation domain (Figure 1A). We expressed these two constructs separately in bacteria, and purified them by tag-affinity chromatography (Figure 1B). Superose 12 gel filtration of Klp6_440_His confirmed that it forms homodimers (Figure 1C). A construct equivalent to our Klp6_440_His could rescue an in vivo *klp6* deletion [47], showing that this short Klp6 construct is functionally competent in vivo.

Microtubule sliding assays using polarity-marked taxol-stabilized microtubules assembled from mammalian brain tubulin showed that both Klp5_436_GST and Klp6_440_His are plus end directed motors (Movies S1 and S2). Sliding velocities for GMPCPP stabilized *S. pombe* microtubules were 6.5±3.6 nm s^−1^ with Klp5_436_GST and 23±12 nm s^−1^ with Klp6_440_His (Table 1, Figure S2). These velocities predict that in vivo, Klp5 and Klp6 will be too slow to keep pace with the plus ends of interphase microtubules growing at ∼50 nm s^−1^ [48]. Klp6 could slide taxol-stabilized brain microtubules at 87 nm s^−1^ (Table 1), showing that our preparation can in principle move faster. Our value for Klp6 on brain microtubules is on the order of the fastest reported velocity for a kinesin-8 of 174 nm s^−1^ for Kif18a [13] (Table S2). Despite Klp5 having about 4-fold slower microtubule sliding velocity than Klp6, the V_max_ and K_m_ for the *S. pombe* microtubule-activated ATPases of Klp5_436_GST and Klp6_440_His are similar (Table 2).

**Table 1:** Microtubule gliding assays.

	Pig brain taxol MTs (nm s^−1^)	*S. pombe* GMPCPP MTs (nm s^−1^)	Pig brain GMPCPP MTs (nm s^−1^)
**Klp6_440_His**	87±18 (1085)	23±12 (77)	56±19 (178)
**Klp5_436_GST**	6.6±3.7 (311)	6.5±3.6 (115)	Not determined

Microtubules were assembled from pig brain tubulin and stabilised with Taxol or *S. pombe* tubulin stabilised with GMPCPP. The velocity of microtubule gliding over surfaces coated with either Klp6 or Klp5 was determined at 25°C. Velocities are mean ± Standard deviation (n).

**Table 2:** Microtubule and tubulin stimulated Klp5/6 ATPase activity.

		Microtubules		Tubulin heterodimer	
		V_max_ (s^−1^)	K_m_ (nM)	V_max_ (s^−1^)	K_m_ (nM)
**Pig brain**	**Klp5_436_GST**	2.3±0.1	27.8±4.9	0.36±0.03	88±26
	**Klp5_436_**	3.1±0.2	45±11	0.36±0.04	427±120
	**Klp6_440_His**	6.3±0.6	85±26	0.032±0.004	337±123
***S. pombe***	**Klp5_436_GST**	3.4±0.2	45±11	0.49±0.04	108±40
	**Klp5_436_**	5.0±0.1	50.0±4.6	0.6±0.1	336±145
	**Klp6_440_His**	2.5±0.2	56±14	0.097±0.009	373±87

Microtubules were assembled from either pig brain tubulin and stabilised with Taxol or *S. pombe* tubulin stabilised with GMPCPP. Microtubule or tubulin heterodimer stimulated ATPase activities were determined in linked assays at 25°C. Non-linear fits of plots of ATPase activity against tubulin concentration were used to determine Vmax and Km values ± standard error of mean.

### Multivalent Klp6 beads are processive

**Figure 2:**
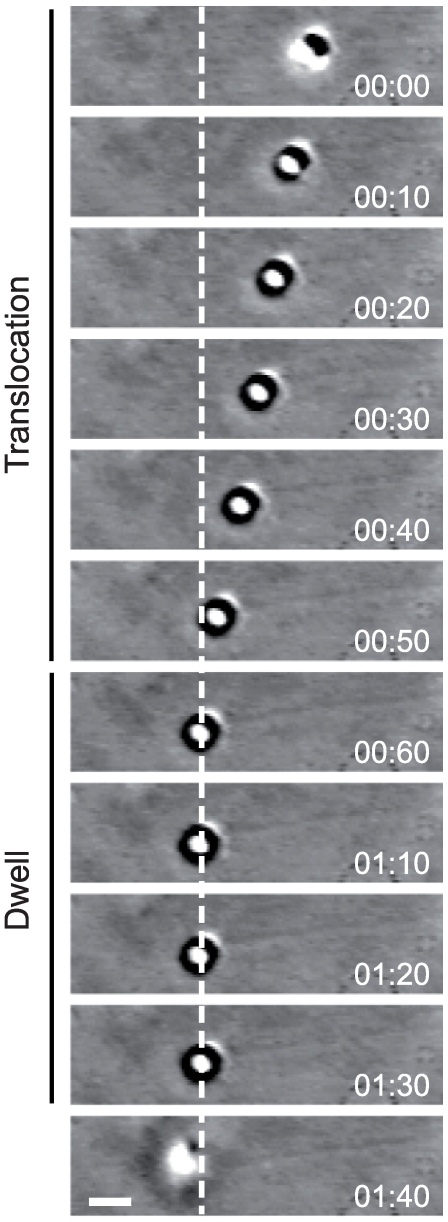
Klp6_440_His coated beads move processively on microtubules. Video-Enhanced Differential Interference Contrast microscopy showing a Klp6_440_His coated bead moving along a taxol-stabilised pig brain microtubule adsorbed to a coverslip. Bead velocity was 47±7 nm s^−1^ (5) (mean ± SEM (n)) during translocation. At the microtubule tip (dashed line) the bead dwelled for several seconds before detaching. Time (minutes : seconds), scale bar:1 µm.

To determine if Klp6 homodimers can move processively, we used beads coated with Klp6. An optical trap was used as a micromanipulator to place Klp6 beads on to immobilized microtubules. Beads carrying multiple copies of Klp6_440_His translocated smoothly towards the plus ends of taxol-stabilized brain microtubules at 42±10 s (6) nm s^−1^ (Figure 2, Table S3). On reaching the microtubule plus ends, the Klp6_440_His beads dwelt for 42±24 s (mean ± SD, n = 6) before dissociating (Figure 2, Table S3). Beads prepared at lower concentrations of Klp6_440_His failed to attach to microtubules. We obtained similar results in microtubule sliding assays on surfaces coated with Klp6_440_His. Reducing the motor density on the surface caused an abrupt transition from smooth microtubule gliding without angular fluctuations at a motor density of 6, 900 µm^−2^ to no microtubules attaching to the surface at 5, 500 µm^−2^. With a kinesin-1 motor rkin430 [49] at a surface density of 300 µm^2^ microtubule movement with angular fluctuations and detachment at microtubule ends was observed, consistent with microtubules sliding over a low density of processive motors [50], [51]. These data suggest that unlike Kip3, Klp6_440_His homodimers are nonprocessive and need to operate in a team of linked multiple motors in order to move processively along microtubules, as has been observed with nonprocessive kinesin constructs attached to beads [52], [53].

### Klp5 and Klp6 do not affect the in vitro dis-assembly of GMPCPP-brain microtubules

Klp5 or Klp6 deletion strains of *S. pombe* contain unusually long microtubules [36], [37], suggesting that Klp5 and 6 destabilise microtubules. To test this hypothesis, we looked for effects of Klp5 and Klp6 on microtubules in vitro. Kip3 is known to accelerate the off-rate of tubulin from GMPCPP-stabilised brain microtubules [10], [14] but it is unclear if this occurs with other kinesins-8 [11], [28]. We found no significant effect of either Klp5_436_GST or Klp6_440_His or combinations of them on the shrinkage rate of pig brain GMPCPP-microtubules by direct observation in the microscope, by light scattering in solution, or in quantitative sedimentation assays (Figures S3, S4, S5, Table S4). Under the same conditions, our positive control, the bona fide depolymerase MCAK, did show a robust depolymerase activity (Figure S3, S5b). We conclude, in agreement with observations on complexes of full length Klp5/6 [12], that Klp5_436_GST and Klp6_440_His do not accelerate depolymerisation of GMPCPP stabilised brain microtubules in vitro.

### Klp5 and Klp6 do not affect the in vitro dynamic instability of S. pombe microtubules

There are no reports on the influence of kinesin-8 motors on dynamically unstable microtubules in vitro. We used direct observation of dynamically unstable, unlabelled *S. pombe* microtubules to assess the effect of Klp5 and Klp6 on microtubule dynamics. In extensive experiments, we found no significant effect on microtubule growth, shrinkage, catastrophe or rescue at either the fast or slow growing ends of dynamic *S. pombe* microtubules (Figures S6, S7, S8, Tables S5, S6, S7, S8, S9, S10). The only exception was Klp5_436_GST, which at very high concentrations corresponding to almost a 1∶1 molar ratio of motor heads to tubulin heterodimers caused a reduction in the shrinkage rate at the faster growing (plus) end (Table S9). Over-expression of a construct with two Klp5 heads in vivo produced a similar effect [38] suggesting that Klp5 activity in our in vitro assays is similar to its activity in vivo at high expression levels. Intriguingly, Kip3 at physiological levels also slows shrinkage in vivo [9].

### Klp6 forms stable complexes with tubulin heterodimers

**Figure 3:**
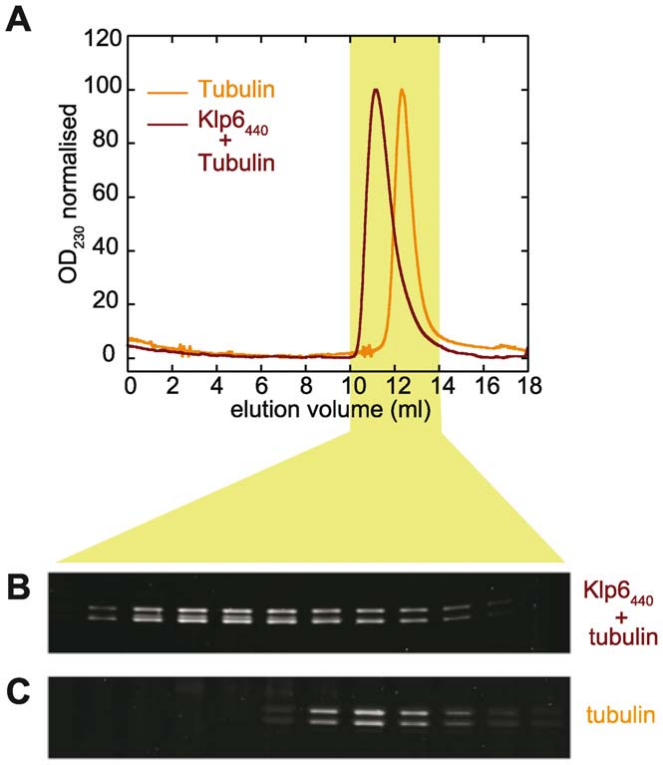
Klp6_440_His forms stable complexes with tubulin heterodimers. (A) Superimposed elution profiles of *S. pombe* single isoform (α1β) tubulin alone (orange) or after incubation with Klp6_440_His (red) from gel filtration on a Superose12 column in buffer containing 0.2 mM GMPCPP. Column fractions from each run were separated by SDS-PAGE before staining and quantification. (B) Fractions from gel filtration of tubulin premixed with Klp6_440_His. (C) Fractions from gel filtration of tubulin alone. In the tubulin only experiment (C) there are two protein bands corresponding to α1 tubulin (upper) and β tubulin (lower). The earlier eluting fractions from gel filtration of the pre-incubated tubulin and Klp6_440_His mixture (B) also contain a third intermediate protein band corresponding to Klp6 between the α1 and β tubulin bands.

We examined the ability of Klp6_440_His to form complexes with tubulin heterodimers using gel filtration chromatography. In the presence of the slowly hydrolysable ATP analogue AMPPNP the single protein peak eluted earlier from the column than with tubulin alone (figure 3a). SDS-PAGE analysis of the eluted fractions showed that in addition to the α1 and β *S. pombe* tubulins, Klp6_440_His was also present in the peak (figure 3b). Quantification of the fluorescently stained gels and correction for differences in the protein staining showed that Klp6_440_His formed a stable complex with tubulin heterodimers with ∼1.0 tubulin heterodimer bound to each kinesin head in the Klp6_440_His homodimer.

### Tubulin heterodimers competitively inhibit the microtubule stimulated ATPase of Klp5 and 6

**Figure 4:**
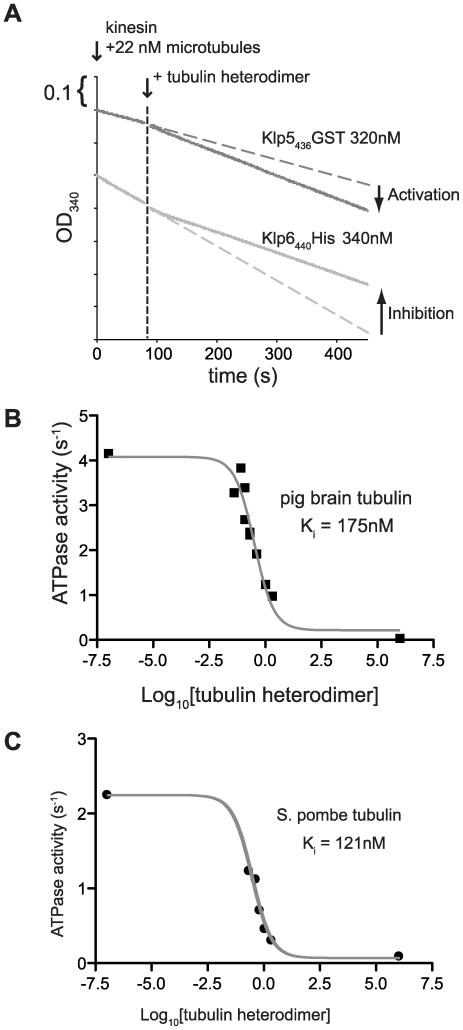
Enzyme-linked assay of competition between tubulin-activated and microtubule-activated ATPases of Klp5_436_GST and Klp6_440_His. (**A**) Mixing kinesin and microtubules at t = 0 gives a constant rate of microtubule-activated ATPase activity (linear slope). Addition of nonpolymerised tubulin (arrow) causes either increased activity for Klp5_436_GST (increased slope) or inhibition of ATPase activity for Klp6_440_His (decreased slope). Klp6_440_His ATPase activity with 73 nM Taxol stabilised pig brain microtubules and increasing concentrations of either (**B**) pig brain or (**C**) *S. pombe* tubulin heterodimers.

To learn more about the mechanisms by which Klp5 and Klp6 interact with tubulin and microtubules, we examined the tubulin-activated and microtubule-activated ATPases. Grissom [12] found that full length Klp5/6 had no tubulin-activated ATPase. We confirmed this is also so for full length Klp6 (Table S1). However, our truncated constructs Klp5_436_GST and Klp6_440_His do have tubulin-activated ATPases (Table 2), suggesting that full length Klp5/6 self-inhibits. Kip3 and Kif18a have substantial tubulin-activated ATPase activity and bind more tightly to tubulin than to microtubules [9] [11]. In contrast Klp5_436_GST and Klp6_440_His bind more tightly to *S. pombe* microtubules than to *S. pombe* tubulin (Table 2). The V_max_ for Klp5 ATPase is 8 fold lower with tubulin than with microtubules whilst the V_max_ for tubulin activation of Klp6 is 250 fold lower than with microtubules. These values predict that in a solution of dynamic microtubules, Klp5 and Klp6 will proportionate between the tubulin-bound and microtubule lattice-bound pools. In assays of microtubule stimulated ATPase activity, addition of tubulin heterodimers enhances the total ATPase activity of Klp5_436_GST, whilst addition of tubulin heterodimers inhibits the total ATPase activity of Klp6_440_His (fig. 4a), with K_i_ of 175 nM for pig brain and 121 nM for *S. pombe* tubulin (Figure 4B, C). This inhibition can be explained by sequestration of Klp6_440_His from the microtubule lattice by free tubulin heterodimers. The re-proportionation of Klp6_440_His between lattice-bound and tubulin-bound pools requires only a few tens of seconds to complete following a perturbation (Figure S10).

### Klp5 and Klp6 drive nucleation of new *S. pombe* microtubules in vitro

**Figure 5:**
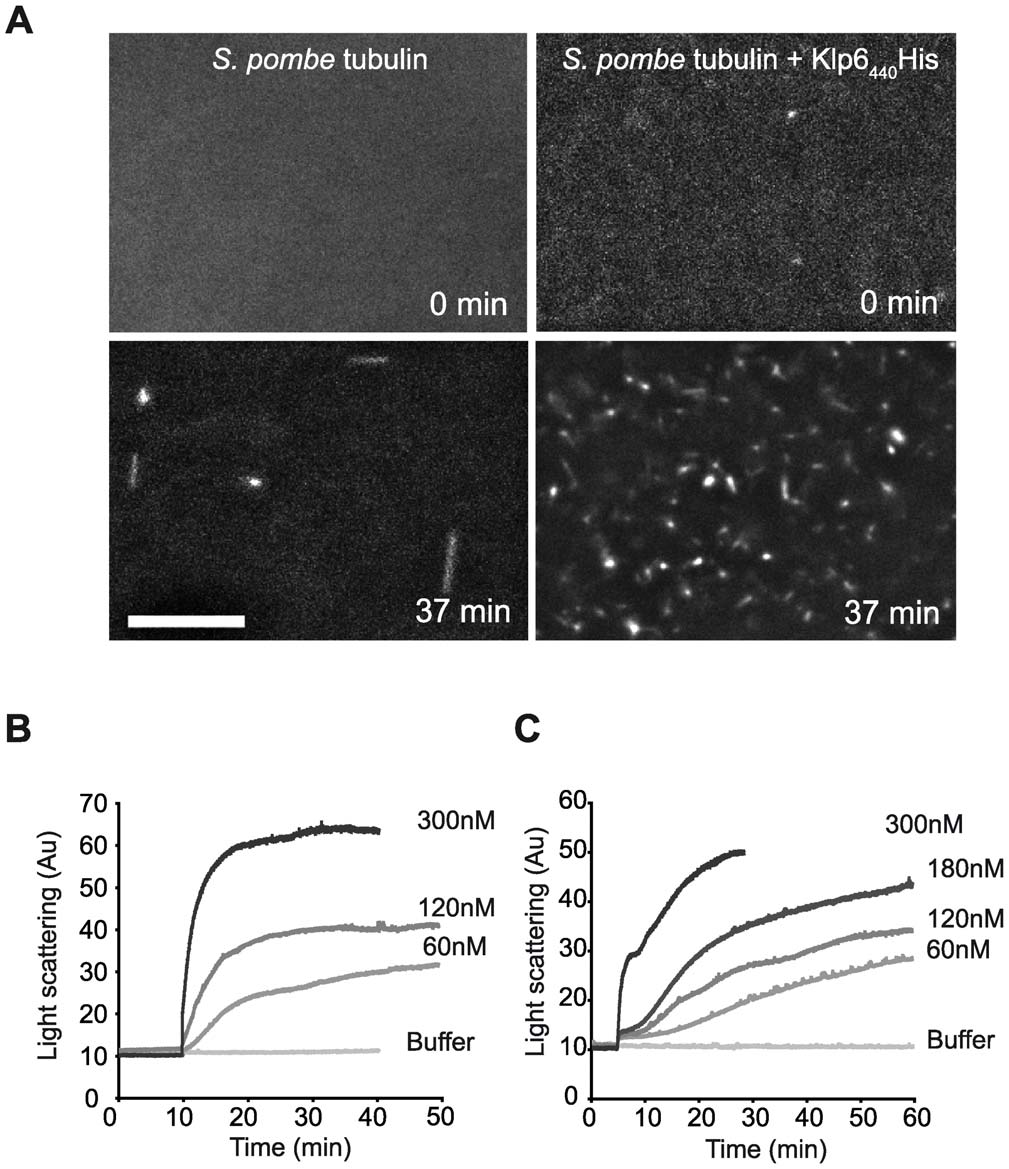
Klp6_440_His enhances tubulin nucleation. (**A**) Polymerisation of 1.5 µM *S. pombe* GMPCPP tubulin visualised by dark field microscopy before and 37 min after adding either buffer or Klp6_440_His. Compared to buffer alone (left) addition of Klp6_440_His drives formation of many short microtubules, indicating a nucleation rather than elongation effect. The effect of Klp6_440_His on the assembly of (**B**) 1.5 µM *S. pombe* or (**C**) 1.5 µM pig brain GMPCPP tubulin into microtubules at subcritical concentration was monitored by light scattering at 350 nm. Increasing Klp6_440_His accelerates the rate of increase in light scattering. Dark field microscopy suggests the increase results from higher microtubule numbers due to increased microtubule nucleation (scale bar: 10 µm).

**Figure 6:**
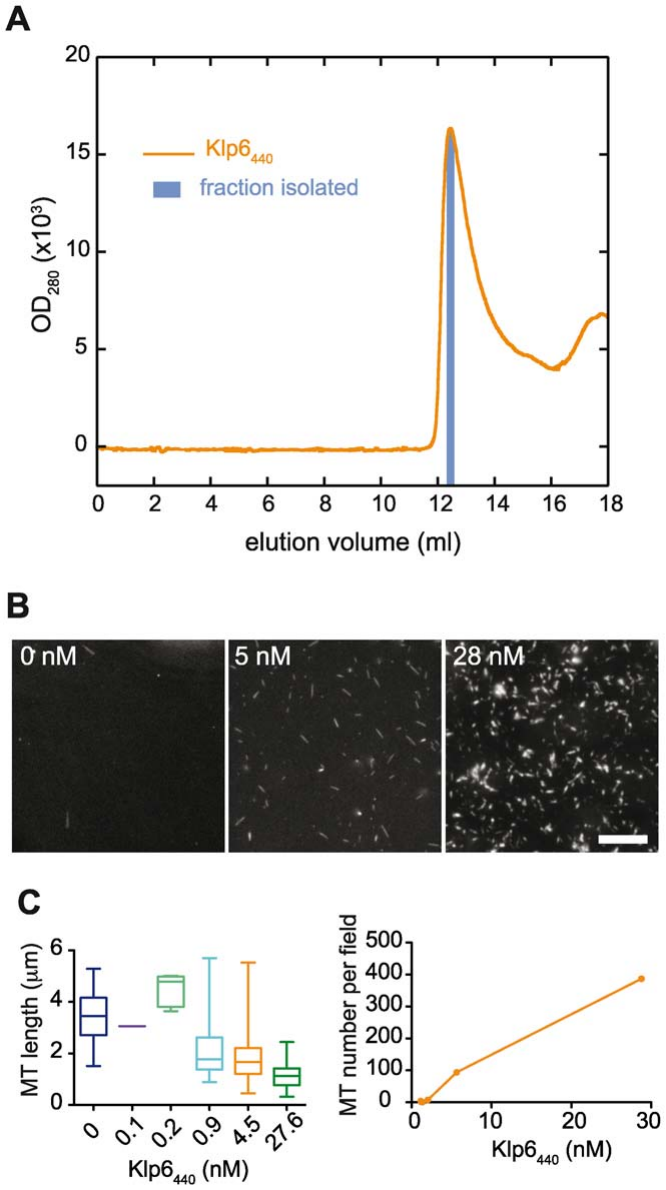
Gel filtered Klp6_440_ promotes microtubule nucleation. A fraction from the single protein elution peak of Klp6_440_His gel filtered on a Superose 12 column (**A**) was immediately incubated with 1 µM *S. pombe* tubulin in buffer containing 1 mM GMPCPP at 25°C. (**B**) Aliquots of samples containing 0, 5 and 28 nM Klp6_440_His were analysed by darkfield microscopy (Scale bar: 10 µm). Microtubule lengths and numbers were measured and plotted against Klp6_440_His concentration (**C**). Increasing Klp6_440_His concentration caused an increase in microtubule number and decrease in microtubule length.

Whilst the in vitro dynamic instability parameters of *S. pombe* microtubules were unaffected by Klp5 or Klp6, it was clear from our experiments that Klp5 and 6 motors very strongly stimulated the formation of microtubules in free solution, independent of the axoneme nucleation centres used in these experiments (Figure S6c, S7c, S8d). We investigated this effect further using GMPCPP to stabilise microtubules under conditions where nucleation is limiting. Addition of Klp6_440_His under these conditions caused the formation of numerous short microtubules (Figure 5a), at a rate dependent on the Klp6_440_His concentration (Figure 5b, c). We could mimic the effect of Klp6_440_His by adding stabilized seeds (very short pre-formed microtubules) to a duplicate sample of tubulin (data not shown), indicating that Klp6_440_His stimulates microtubule assembly by inducing the formation of nuclei at tubulin concentrations where nucleation is normally limited. Klp5 had a similar, though less potent, effect (Figure S8). Both motors promoted microtubule nucleation in both the presence and absence of ATP (not shown). To ensure the nucleation effect was not caused by protein aggregates that might form during freezing and storage of the proteins, Klp6_440_His was gel filtered and an aliquot taken from the peak of monodisperse protein (fig. 6a). This repurified protein produced both a dose-dependent increase in the number of microtubules formed from *S. pombe* tubulin and a dose-dependent reduction in mean microtubule length (fig. 6b, c), consistent with it promoting microtubule nucleation.

We considered two possible classes of nucleation mechanism. First, Klp5 and Klp6 binding to tubulin heterodimers might alter their conformation to favour assembly. Second, dimeric Klp5 and Klp6 might link tubulin heterodimers together to stabilise nascent microtubule nuclei. To distinguish these possibilities, we tested Klp6_415_His, a single-headed Klp6 construct (fig. 1c), which has microtubule stimulated ATPase activity similar to Klp6_440_His. Klp6_415_His did not detectably enhance microtubule nucleation (data not shown), showing that the binding of Klp6 heads to free tubulin does not in itself drive microtubule nucleation. Instead, enhancement of nucleation requires a dimeric construct that can then link two tubulin heterodimers.

We also found that double headed Klp5 and Klp6 constructs, either individually or when co-expressed, could crosslink microtubules and cause bundling of both dynamic microtubules (Figure S6c, S7c, S8d) and of stable preformed GMPCPP-microtubules (Figure S9), similar to the bundling reported for full length Klp5/6 [12]. Bundling was absent when microtubules were assembled by adding Klp5/6 to free tubulin (Figure 5, 6), possibly because both tubulin binding sites are then occupied, leaving no sites free to support microtubule cross linking. Single-headed Klp6_415_His does not drive bundling (Figure S5) suggesting that microtubule cross-linking occurs by the same mechanism that permits binding of two heterodimers.

### Klp5 & Klp6 stimulate microtubule catastrophes in vivo

**Figure 7:**
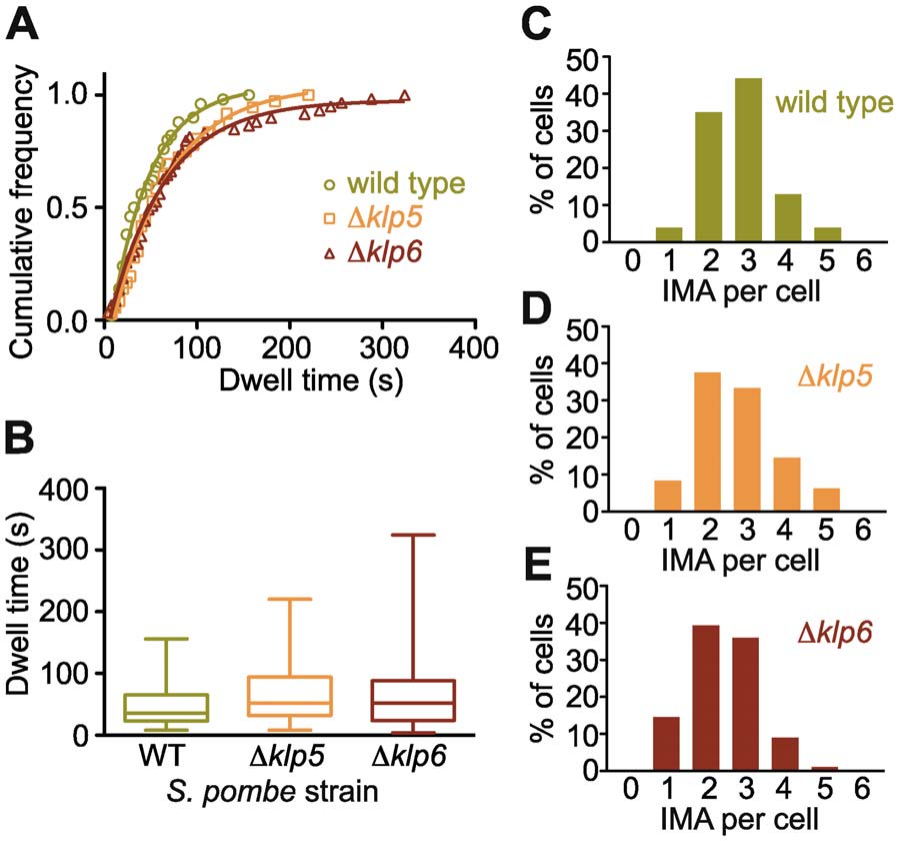
Deletion of *klp5* or *klp6* increases microtubule dwell time in vivo. Time-lapse movies (Movies S3, S4, S5, S6) of GFP-microtubules in live wild type (wt), *klp5* or *klp6* deletion mutant *S. pombe* were analysed and the dwell time of microtubules in contact with the cell wall before undergoing catastrophe recorded. (**A**) Cumulative frequency plots of dwell times were each fitted with a single exponential. The half-lives of the dwell time were significantly longer in Δ*klp5* (42 s) and Δ*klp6* (42 s) cells compared to wild type (29 s, p<0.05). (**B**) Box and whisker plots for the microtubule dwell time data plotted in (A) showing median and interquartile range (box) and distribution limits (whiskers). Median values are wt: 36 s, Δ*klp5*: 52 s and Δ*klp6*: 52 s. Deletion of *klp6* decreases IMA number in vivo. Normalised frequency distribution plots for IMA number per *S. pombe* cell in wild type (WT, **C**), Δ*klp5* (**D**) and Δ*klp6* (**E**) strains. Mean numbers of IMA per cell were wild type: 2.8±0.1 (mean ± SEM, n = 77); *klp5* deletion: 2.7±0.1 (n = 48); *klp6* deletion: 2.4±0.1 (n = 89). The reduction in IMA number in *klp6* deletions compared to wild type is significant with P = 0.01.

To learn more about the mechanisms of full length Klp5/6 in vivo and in context, we revisited live cell imaging of *Klp5/6* deletion mutants, taking data at higher time resolution than has previously been achieved (Movies S3, S4, S5). Catastrophe events in wild type *S. pombe* cells occur almost entirely in the end zone of the cell [43], [48], [54]. Figure 7A is a cumulative frequency plot showing the fraction of cell-end-resident microtubule tips that undergo catastrophe within a specified period. The data reveal a clear, statistically significant difference between microtubule lifetimes in the end zone of wild type cells and Δ*klp5* cells, and between wild type and Δ*klp6*. In both deletion strains, the half-life (the time for 50% of microtubules in contact with the cell end to undergo catastrophe) increased from 29 to 42 seconds (p<0.05). The median dwell time of microtubule tips at cell ends increased from 36 seconds in wild type to 52 seconds in both Δ*klp5* and Δ*klp6* (Figure 7B). A similar trend was reported by Unsworth et al. [38]. We thus confirm that in vivo and within the context of the end zone of wild type *S. pombe* cells, Klp5 and Klp6 are accelerators of microtubule catastrophe.

### Deletion of Klp6 reduces microtubule number in vivo

We found that deletion of *klp6* also causes a reduction in the number of interphase microtubule assemblies (IMAs [48]) from 2.8±0.1 (mean ± SEM, n = 77) in wild type to 2.4±0.1 (n = 89) in *klp6* deletions (P = 0.01), whilst deletion of *klp5* had no significant effect (2.7±0.1, n = 48) (Figure 7c, d, e). This reduction in IMA number in vivo is consistent with our observation that Klp6 had a strong nucleation effect in vitro whilst the effect of Klp5 was weaker. IMAs normally have a bipolar microtubule arrangement so that microtubules depolymerise to the centre of the cell [48], [55], [56]. However in Δ*klp6* mutants we found that some depolymerisations continued along the entire cell length (Movie S6), suggesting a disrupted IMA structure with unipolar microtubules as observed in Δ*klp2* [57].

## Discussion

### Klp5/6 promote microtubule depolymerisation in vivo but not in vitro

Our observations show that Klp5/6 destabilise interphase microtubules in *S. pombe* cells by reducing the time between the growing microtubule contacting the cell end and undergoing catastrophe, similar to effects reported in previous studies [38], [43]. What remains unclear is the underlying molecular mechanism. Acceleration of GMPCPP-stabilised microtubule depolymerisation in vitro by Kip3 [9], [10] has led to the working assumption that all kinesins-8 would act on microtubules by a similar mechanism. However demonstration of a Kip3-like destabilisation of microtubules in vitro by other kinesins-8 is limited to a single report for Kif18a [11]. Subsequent studies have found no effect of either Kif18a [28] or Klp5/6 [12] on in vitro microtubule stability.

A common feature of microtubule depolymerases is tubulin heterodimer stimulated ATPase activity [9], [58], which was absent in the full-length constructs of Klp5/6 used in previous studies [12]. We confirmed that full length Klp6 lacked tubulin stimulated ATPase but found that the truncated tail-less Klp5 and Klp6 did show tubulin-activated ATPase, suggesting that full length Klp5/6 may auto-inhibit. A Klp6 truncation equivalent to ours can rescue the loss of microtubule destabilising activity in *klp6* deletions [47], demonstrating that the truncated construct is functionally competent in restoring normal microtubule lengths in vivo. We find that despite having microtubule translocase activity, neither full length nor truncated Klp6 constructs promote depolymerisation of GMPCPP brain microtubules, or of GMPCPP *S. pombe* microtubules. Even on dynamic *S. pombe* microtubules, no significant effect on microtubule stability was observed using truncated Klp5 and 6 constructs alone or in combination, despite all of the constructs having active ATPase and microtubule translocase activity. Translocase activity was observed directly in the assays confirming the presence of active kinesin. We conclude that Klp5 and Klp6 lack an in vitro microtubule depolymerase activity.

### Klp5/6 in vivo will only keep pace with growing MT tips at cell ends

Klp6 and Klp5 translocate *S. pombe* microtubules in vitro at 23 nm s^−1^ and 7 nm s^−1^ respectively. Kip3 moves with similar velocities in vitro and in vivo [9], [10] at about twice the rate of microtubule elongation in vivo [9], [15], which is sufficient to account for the accumulation of Kip3 at growing microtubule ends by translocation along the microtubule. If the velocity of Klp5 and Klp6 in vivo is similar to their microtubule sliding velocities in vitro they would be too slow to keep pace with the microtubule tip growing at 35–65 nm s^−1^ in *S. pombe* [38], [43], [48], [55], [59], [60], [61], [62]. This implies either that a factor exists in vivo which accelerates the Klp5/6 microtubule translocase activity above the velocity observed in vitro, or that Klp5/6 track microtubule ends by a different mechanism. We also found in both microtubule sliding assays and with Klp6 coated beads moving along microtubules that a double headed Klp6 motor domain construct at low densities is non-processive. Only when moving in multiple motor teams is processive movement along microtubules possible, as observed with other non-processive kinesin constructs [52], [63]. Redistribution in 10–20 seconds between microtubules and tubulin heterodimers during ATPase assays also suggests that both Klp5 and Klp6 motor domains are either non-processive or have very limited processivity. At present it is unclear if Klp5 and 6 have a microtubule binding region in their tail domain as found in Kip3 and Kif18a [13], [26], which might confer processivity on full length proteins.

Non-processivity of Klp5/6 would rule out a Kip3-like mechanism, which requires high processivity coupled to a translocation velocity that is higher than the microtubule growth rate [10], [14]. However Klp5/6 has been shown to accumulate at the ends of growing microtubules in vivo [43]. Since we observed that Klp5/6 motor domains could bind tubulin heterodimers and form stable complexes we propose that Klp5/6 could accumulate at microtubule ends in complex with tubulin heterodimers as they incorporate at the microtubule tip rather than by procession along the lattice. After incorporation the Klp5/6 complex would be left behind by the growing microtubule tip and the motor would rapidly dissociate from the microtubule lattice. This cycle can form the basis of a tip-tracking mechanism. In vivo, tip tracking is likely to involve other proteins: for example Alp14, a member of the TOG/XMAP215 family of tip-tracking proteins has a role in *S. pombe* in localising Klp5 to kinetochores [40].

### Klp5/6 drives MT nucleation in vivo and in vitro

In cells Klp5/6 promote microtubule growth, catastrophe and rescue, thereby increasing overall microtubule dynamicity [38]. We observed a significant reduction in the IMA number in cells lacking Klp6 suggesting a role for Klp6 in IMA formation or stabilisation. No effect on IMA number was observed in *klp5* deletions. This also shows that the Klp6 effect does not simply arise as a consequence of the altered free tubulin level in the deletion strain since *klp5* and *klp6* deletions both affect microtubule catastrophe frequencies yet only *klp6* deletions had altered IMA numbers. Some of the IMAs formed in *klp6* deletions had abnormal monopolar arrangements of microtubules previously only observed in deletions of *klp2* [57], consistent with Klp6 influencing IMA formation.

In vitro we found that Klp5/6 promotes microtubule nucleation without increasing growth rate. This is a hitherto-undescribed activity for kinesin motors. *Xenopus* XKlp1, a kinesin-4, has microtubule-stabilizing activity [64], but also inhibits growth and shrinkage, whereas Klp5 and Klp6 do not affect growth and Klp5 only affects shrinkage at very high concentrations. Our data suggest that Klp5/6 homodimers may promote microtubule nucleation by cross-linking tubulin heterodimers. Potentially, Klp5/6 might grip the lattice of nascent microtubule nuclei and link the subunits together, inhibiting subunit dissociation until a stable microtubule structure forms. Our data show that the lattice-bound Klp5 and Klp6 molecules then dissociate and exchange back into the free tubulin pool within 10–20 seconds.

### A new working model for the in vivo mechanism of Klp5/6

**Figure 8:**
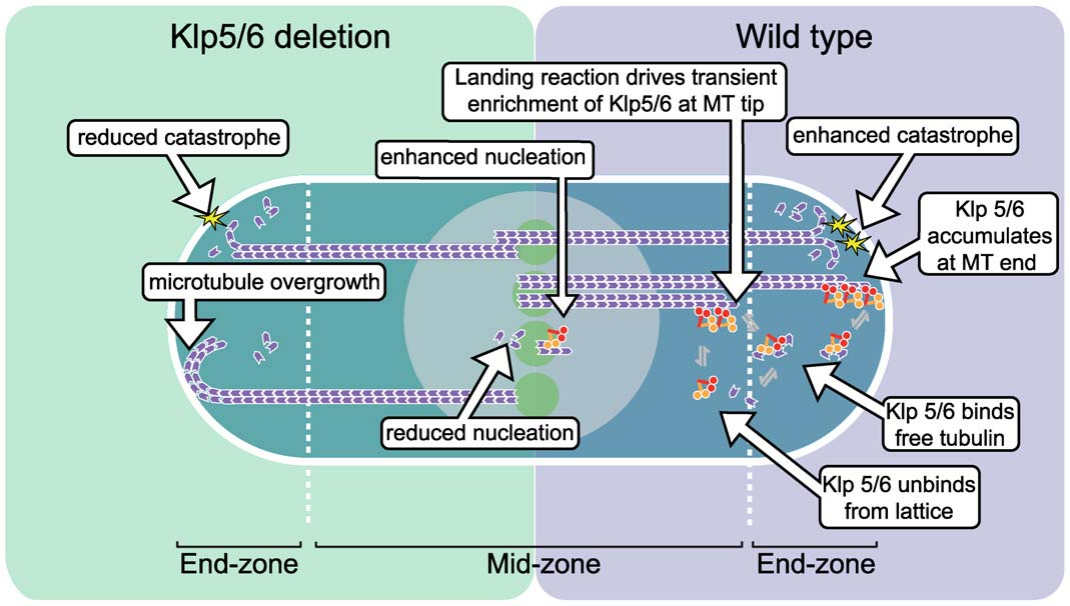
Model for the role of Klp5/6 in microtubule dynamics in vivo. Cartoon of an interphase *S. pombe* cell with the nucleus (grey disc) and microtubule nucleation sites (green discs) in the cell centre. The right-hand side shows wild type cells with microtubules (purple) nucleating at the cell centre, growing into the end zone of the cell and undergoing catastrophe in contact with the cell end. Klp5/6 (turquoise/red) heterodimers help promote nucleation, crosslink microtubules in the IMAs and accelerate catastrophe at the cell end, possibly by physical linkage of the microtubule tip to the cell cortex. Klp5/6 continuously incorporate at the microtubule tip but rapidly detach as the tubulin dimers are incorporated into the microtubule lattice. Thus, Klp5/6 continuously sample the location of the microtubule within the cell. Microtubule growth slows after the microtubule contacts the cell end, allowing accumulation of Klp5/6 by incorporation and short-range translocation. The left-hand side shows a *klp5/6* deletion mutant, in which the absence of Klp5 or Klp6 reduces nucleation activity, weakens cross-linking in the IMAs and increases the dwell time of the microtubule tips at the cell end before they undergo catastrophe, leading to some microtubule overgrowth.

A model for Klp5/6 activity in vivo has to account not only for microtubule destabilisation activity but also for other apparently contradictory roles including both enhanced microtubule rescue [38] and enhanced IMA formation. Our observations on Klp5/6 are incompatible with MCAK-like or Kip3-like mechanisms, in which the motor depolymerises the GTP cap of dynamic microtubules in solution. Instead, we favour a model in which Klp5/6 are dynamases that first promote the birth of new microtubules, and then shorten their lifetime by accelerating catastrophe in the end zone of the cell (figure 8). In our model, Klp5/6 activity promotes the formation of new microtubules. Thereafter, Klp5/6 will continuously land on the growing microtubule tip as a complex with GTP-tubulin heterodimers, consistent with the known tendency of Klp5/6 to enrich at microtubule tips [43]. The GTP-tubulin will incorporate into the microtubule lattice and Klp5/6 will dissociate after a few seconds of residence time. According to our data, Klp5/6 is not fast enough to keep pace with the growing tip. Furthermore dimers of Klp5 or Klp6 are non-processive and would only be able to translocate along the microtubule if they assemble into a multiple motor complex. Only at the cell ends, where the microtubule tip engages with the inner cell membrane and its growth rate approximately halves [48], [55] do we predict that Klp6 motility would be similar to that of the microtubule tip and Klp6 would have the potential to enrich more substantially than during microtubule growth through the cytoplasm. The extent of enrichment by translocation along the microtubule would depend on formation of multimotor complexes or attachment of the motors to other proteins that might enhance processivity. Our data show that Klp5/6 does not cause catastrophe of dynamic microtubules in vitro whereas Klp5/6 in its natural in vivo environment does accelerate microtubule catastrophe. Clearly the cellular context is required for the catastrophase activity of Klp5/6. It is possible that our Klp5/6 constructs lack an activating post-translational modification or binding partner found in cells. However, we doubt this is the case since our constructs are active, in that they have microtubule and tubulin stimulated ATPase activity and can translocate microtubules. Instead, we suggest that the missing factor in our in vitro assays is mechanical compression of the microtubule tip. In *S. pombe*, microtubule catastrophes occur almost exclusively in the end zone of the cell, with the microtubule tip in contact with the cell wall and in compression [43], [48], [54]. Mechanical force can trigger microtubule catastrophe in vitro [65] and a role for force has been suggested in *S. pombe* [44], [48]. The growing plus end of a dynamic microtubule is thought to carry a small sheet of protofilaments that has yet to close into a tube, that may resemble the nucleus formed at the outset of microtubule assembly [66]. We speculate that Klp5 and Klp6 would stabilise the sheet in the absence of external forces by linking heterodimers, thus promoting nucleation. However at cell ends Klp5 and 6 would no longer stabilise but rather destabilise the microtubule. This might occur through the same property of gripping the tip-sheet if Klp5/6 were, for example, to link to the cell end and apply enhanced force to the already compressed microtubule. We propose that the effect of this additional destabilising force exerted by Klp5/6 on the microtubule is sufficient to overcome the stabilising effect of Klp5/6 binding, further increasing the catastrophe frequency.

Our working model for Klp5/6 is part substance and part speculation. As in the Kip3 model [43], [44], catastrophe is microtubule length-dependent, but in our model control of microtubule length is achieved by amplifying the intrinsic tendency for microtubules to catastrophise in compression in the end zone of the *S. pombe* cell. Testing these proposals will require the development of improved in vitro reconstitution systems that include other microtubule or Klp5/6 interacting proteins, and can determine the influence of mechanical force on microtubule dynamics.

## Materials and Methods

### *S. pombe* strains

*S. pombe* strains were kindly given by T. Toda, [37] Δ*Klp5*: NK006 (*h^+^ leu1.32 ura4.d18 klp5::ura4^+^ kan^r^-nmtP3-GFP-atb2^+^*), Δ*Klp6*: NK007 (*h^−^ leu1.32 ura4.d18 klp6::ura4^+^ kan^r^-nmtP3-GFP-atb2^+^*) and wild type wt: *GFP-nmt1-atb2 (h^−^ leu1.32 kan^r^-nmt1-GFP-atb2^+^)*.

### Plasmid Constructs

Plasmid pET-Klp6_440_His encoding the protein Klp6(aa1–440)/GlyGly/6×His tag (Klp6_440_His) was created by PCR amplification (Phusion polymerase, Finzymes) from cosmid c649 (Sanger Centre, [67]) using primers klp6f1/r2 (Table S11). The DNA fragment was cloned into the BamHI/XhoI sites of pET-Klp6_1–415_ (encoding amino acids 1–415, a kind gift from Aini Hamid and Linda Amos, unpublished). Plasmid pET-KLp6_FL_His, encoding protein Klp6(aa1–784)/GlyGly/6×His tag (Klp6_FL_His) was created by cloning the BamHI/SmaI digested PCR product from c649 and primers klp6f1/r3 (Table S11) into the BamHI/EcorV sites of pET17b (Novagen). This construct was digested with NdeI and BamHI, and then the NdeI/BamHI fragment from pET-Klp6_440_His was inserted. Plasmid pET-His-Klp6_FL_ encoding 6×His tag/GlyGly/Klp6(aa1–784) was generated by PCR of pET-KLp6_FL_His using primers klp6f4/r2 (Table S11). The product was NotI digested, end filled by Klenow fragment polymerase, NdeI digested and cloned into the NdeI and EcoRV sites of pET17b. Plasmid pET-Klp5_436_GST encoding Klp5(aa1–436)/PreScission cleavage site/GST was created by PCR amplification of cosmid c2F12 (supplied by Sanger centre, [68]) of two overlapping fragments using primer pairs klp5f2/r4 and klp5f3/r5. A further PCR using klp5 f1/r5 fused the two fragments, removing the intron and internal NdeI site. The fragment was NdeI/EcoRI digested and cloned into the NdeI/EcoRI sites of pET24b containing an EcoRI/NotI fragment encoding a C-terminal GST tag and stop codon (a kind gift from Maria Alonso, unpublished). All constructs were confirmed by DNA sequencing (Cogenics).

### Protein expression

Proteins were expressed in Rosetta™ 2 (DE3) *E. Coli* (Novagen) grown overnight 37°C, diluted 1∶50 in 2×YT medium containing 34 µg/ml chloramphenicol and either 100 µg/ml ampicillin for Klp6 or 30 µg/ml kanamycin for Klp5 constructs, or all three antibiotics for co-expression. Expression was induced by 1 mM IPTG and incubating for a further 4 h at 25°C.

### Protein purification

Bacterial pellets were re-suspended in column buffer containing 0.5 mM DTT, Complete Protease Inhibitor EDTA Free Cocktail tablets (Roche), made 0.1 mg/ml lysozyme, and incubated on ice 20 min. The lysate was made 0.05% (v/v) Triton X-100, 10 mM MgCl_2_, 40 µg/ml Deoxyribonuclease I, incubated 10 minutes on ice and clarified by centrifugation (47800 g, 4°C, 35 min). Klp6 His-tagged constructs, were loaded on a HisTrap® column (GE Healthcare) in 20 mM K^+^Phosphate pH 7.4, 500 mM KCl, 5 mM Imidazole, 1 mM MgCl_2_, 50 µM ATP, 0.5% (v/v) NP-40. The column was first washed with low salt buffer (20 mM K^+^Phosphate pH 7.4, 150 mM KCl, 1 mM MgCl_2_, 50 µM ATP, 0.5% (v/v) NP-40) containing 5 mM imidazole, then with low salt buffer containing 96 mM imidazole. Proteins were eluted with a 96–500 mM imidazole gradient in low salt buffer. Klp5_436_GST was loaded on a GSTrap® column (GE Healthcare) in PBS (phosphate buffered saline), 1 mM MgCl_2_, 50 µM ATP, 0.5% (v/v) NP-40 and washed with the same buffer. Proteins were eluted in 50 mM Tris-HCl, pH 8.0, 150 mM KCl, 10 mM glutathione, 0.5% (v/v) NP40, 1 mM DTT, 50 µM ATP, 1 mM MgCl_2_. For some experiments the GST tag was removed by PreScission protease cleavage (GE Healthcare). Co-expressed Klp5_436_GST and Klp6_440_His were purified by sequential use of the His tag and GST tag methods.

### Protein storage and quantification

Kinesin-containing fractions were pooled and small aliquots flash-frozen in liquid nitrogen. All preparations were subsequently either desalted into the appropriate buffer using Zeba spin columns (Pierce) or microtubule affinity purified and flash frozen again. Proteins were analysed by SDS-Page (NuPAGE 10% Bis-Tris gels run in MOPS buffer, Invitrogen) stained with either InVision™ His-tag fluorescent dye (Invitrogen) for Klp6_440_His or Sypro Red (Invitrogen) for Klp5_436_GST. Gels were imaged (Molecular Imager FX, BioRad) and concentrations determined by comparison with standards (Quantity one software (BioRad)).

For use in microtubule dynamics assays HisKlp6_FL_, Klp5_436_GST/Klp6_440_His and Klp5_436_GST purified proteins were mixed with a 5 fold molar excess of NV10 (Novexin Ltd) and desalted (0.5 ml Zeba column, Pierce) into K-PEM (100 mM PIPES, 1 mM MgSO_4_, 2 mM EGTA, adjusted to pH 6.9 with KOH [2]) containing 50 µM ATP and 1 mM DTT. Proteins were frozen in small aliquots in liquid nitrogen. Freezing reduced microtubule stimulated Klp6 ATPase activity by 7%. Protein concentration was measured using a calculated ε_280_ [69] of 33190 for His-KLP6_FL_ monomer assuming one ATP per kinesin head, or ε_280_ of 75,340 assuming equimolar concentrations of Klp5_436_GST/Klp6_440_His. Klp5_436_GST OD_280_ was corrected for nucleic acids [70]. Microtubule stimulated ATPase was HisKlp6_FL_, 0.68 s^−1^ head^−1^ (1.99 µM MTs); Klp5_436_GST/Klp6_440_His, 0.07 s^−1^ head^−1^ (1.68 µM MTs).

### Microtubule affinity purification of Klp5 and Klp6

Klp6_440_His or Klp5_436_GST were incubated 10 minutes 25°C with an excess of Taxol (paclitaxel) stabilized pig brain microtubules (typically 7.5 µM), 1 mM AMP.PNP, 20 µM Taxol in BRB80. Microtubules were pelleted at 108, 920 g, 10 min, 25°C (TLA 100 rotor, Optima TLX centrifuge, Beckman), washed with 100 µl of BRB80, 20 µM Taxol then resuspended in 50 µl BRB80, 100 mM KCl, 10 mM MgCl_2_, 10 mM ATP, 20 µM Taxol, 0.5% (v/v) NP-40. Microtubules were pelleted again, the supernatant aliquoted, flash-frozen in liquid nitrogen and samples quantified.

### *S. pombe* microtubule affinity purification of Klp5_436_GST/Klp6_440_His

Klp5_436_GST/Klp6_440_His was incubated with a 5 fold molar excess of NV10 (Novexin Ltd), 5.2 µM *S. pombe* GMPCPP microtubules, 0.53 mM MgAMP.PNP 0.53 mM DTT in K-PEM and processed as before using 20 nM GMPCPP, 1 mM DTT in K-PEM wash before re-suspension in K-PEM, 500 mM K.Acetate, 10 mM Mg.ATP, 20 nM GMPCPP, 1 mM DTT, 1 mg/ml NV10 for 15 min at 25°C. The post-centrifugation supernatant was desalted (Zeba column, Pierce) into 100 µM ATP, 1 mM DTT, 20 µM GDP in K-PEM. Recovery was estimated by SDS-PAGE. Tubulin from the purification co-migrates with Klp6_440_His, so Klp5_436_GST was quantified and Klp6_440_His assumed to be equimolar. Affinity purification increased microtubule stimulated ATPase activity ∼10×. Tubulin concentrations in dynamics assays were adjusted to compensate for tubulin carry over.

### Purification of Tubulin

*S. pombe* tubulin was prepared from the wild type strain of *S. pombe* (972 h^−^) containing the α1β and α2β isoform or a single isoform strain containing α1β isoform tubulin heterodimers as described in [71], [72]. Pig Brain tubulin was prepared as described in [73]. Purified tubulins were desalted into K-PEM buffer (100 mM PIPES, 1 mM MgSO_4_ and 2 mM EGTA adjusted to pH 6.9 with KOH [2]) containing 20 µM GDP before storage in liquid nitrogen. Protein concentrations were determined by OD_280 nm_ in 6 M GuHCl, assuming full nucleotide occupancy and using a calculated extinction coefficient [69] ε = 105838 M^−1^ cm^−1^ for pig brain tubulin and 108,390 M^−1^ cm^−1^ for *S. pombe* tubulin.

### Gel filtration chromatography of Klp6_440_His, Klp6_415_His

Samples containing either Klp6_440_His, Klp6_415_His or a mixture of both were centrifuged in a microcentrifuge (10 min, 4°C, 20870 g) and then directly loaded on a Superose-12 HR 10/300 GL column (GE Healthcare) held at 4°C and running at 0.5 ml min^−1^ with 100 mM PIPES, 200 mM NaCl, 1 mM MgCl_2_, 2 mM EDTA, pH 6.9, in an ÄKTA purifier 10 system (GE Healthcare). For purification of Klp6_440_His the purified protein was first desalted into BRB80 (80 mM PIPES, 1 mM MgCl_2_, 1 mM EDTA, pH 6.9) using Zeba 0.5 ml minispin columns (Pierce) before loading and elution from a Superose-12 column in BRB80 buffer.

### Gel filtration chromatography of Klp6-tubulin complexes

15 µM *S. pombe* single isoform tubulin (α1β tubulin) or mixtures of 15 µM tubulin and 10.7 µM dimeric Klp6_440_His (equivalent to 21.4 µM kinesin heads) in 80 mM PIPES, 2.5 mM MgCl_2_, 1 mM EDTA, 1.5 mM GMPCPP, 1 mM DTT pH 6.9 were incubated at 4°C for 30 min, centrifuged at 20 000 g for 10 min, 4°C then 150 µl of the supernatant separated by gel filtration on a Superose-12 HR 10/300 GL column (GE Healthcare) held at 4°C and running at 0.5 ml min^−1^ with 80 mM PIPES, 1.2 mM MgCl_2_, 1 mM EDTA, 1.2 mM GMPCPP pH 6.9, in an ÄKTA purifier 10 system (GE Healthcare). Samples from column fractions were separated by SDS-PAGE using 4–12% gradient Nu-PAGE gels in MOPS buffer (Invitrogen) run at 200 V for 80 min and visualized by staining with Krypton fluorescent protein stain (Thermo-scientific) and imaged using an Odyssey laser gel scanner (Li-COR Biosciences). Preferential staining of tubulin was corrected by comparison with BSA protein standards.

### Steady state ATPase

The microtubule or tubulin heterodimer stimulated ATPase activity of microtubule affinity purified kinesin was measured at 25°C using a linked assay as described [73] except the PIPES concentration was increased to 80 mM and ATP to 1 mM. Microtubules assembled from pig brain tubulin and GTP or *S. pombe* tubulin and GMPCPP were pelleted and resuspended in BRB80, 20 µM Taxol or BRB80 respectively before dilution into the linked assay buffer (80 mM PIPES, pH 6.9, 5 mM MgCl_2_, 1 mM DTT, 0.1 mg/ml BSA). For measurements with pig brain microtubules 20 µM taxol was added to the buffer. NADH absorption was monitored at 340 nm in a Cary50 spectrophometer (Varian) using 70 µl disposable cuvettes (Eppendorf). Values for V_max_ and K_m_ were obtained by least squares fitting of the Michaelis-Menten equation to plots of ATPase versus tubulin heterodimer concentration using Prism (GraphPad Software Inc.). ATPase values are per kinesin head. IC50 was obtained by fitting the one site competition equation Y = heterodimer V_max_+(MT V_max_−heterodimer V_max_)/1+10^(X-logIC50)^) to the plot of ATPase activity versus tubulin heterodimer concentration using Prism software. IC_50_ is the concentration of Tubulin heterodimer inhibiting 50% of activity. K_i_ is calculated from the IC_50_ value according to [74].

### In vitro motility assay

Pig brain microtubules were stabilised by 20 µM Taxol and *S. pombe* microtubules by 1 mM GMPCPP. Coverslips were coated with anti-His tag antibody (Qiagen) or polylysine and anti-GST antibody (Santa Cruz biotechnology Inc.) then blocked with 5 mg/ml BSA. Microtubule affinity purified kinesins were flushed into the chamber and incubated 10 min, followed by microtubules diluted in 80 mM Pipes, pH 6.9, 1 mM EDTA, 1 mM MgCl_2_, 5 mM DTT, 2 mM ATP. The buffer also contained 20 µM taxol for pig brain microtubules and 50 mM KCl for Klp5_436_GST. *S. pombe* microtubule dilution buffer contained 50 mM KCl for both Klp6_440_His and Klp5_436_GST. Microtubules were visualised by VE-DIC [75] and their velocity analysed using RETRAC software (http://www.mechanochemistry.org).

Microtubule motility assays on surfaces with low kinesin density used affinity purified Klp6_440_His diluted in buffer containing K-PEM, 100 mM KCl, 1 mM ATP, 1 mM MgCl_2_, 0.5% (v/v) NP40, 1 mg/ml BSA then flushed into a flow cell coated with anti-His antibody and incubated for 5 min to allow binding of the motor to the surface. The chamber was about 100 µm deep and complete absorption of kinesin to the surface is expected within a few minutes [50]. The chamber was washed with K-PEM, 1 mM ATP, 1 mM MgCl_2_, 5 mM DTT, 20 µM Taxol. Then the same buffer with 0.05 µM pig brain microtubules stabilised with Taxol. In assays where no binding of microtubules to the kinesin surface was observed non-binding was confirmed by flushing in 0.25 µM pig brain microtubules to increase the probability of microtubules colliding with the few kinesins present. Control experiments used the processive rat kinesin-1 rkin430GST [49] bound directly to the flow cell. Surface motor densities were calculated from the dimer kinesin concentrations in the dilutions and assuming all added motor bound to both the upper and lower flow cell surfaces [50].

### Bead assay

0.5 mg of Streptavidin-coated polymer beads (500 nm diameter, Bang Laboratories Inc.) were mixed with 120 µg of Penta-His antibody (Qiagen) in PBS, 1% (w/v) BSA, 0.05% (v/v) Tween20. After centrifugation (20000 g for 2 min) the beads were re-suspended in 100 µl of PBS, 1% (w/v) BSA, 0.05% (v/v) Tween20.

5 µl of Klp6_440_His from a frozen microtubule affinity purified stock was mixed with 2 µl of beads and 8 µl of BRB80, 1% (w/v) BSA, 0.05% (v/v) Tween20 and incubated for 15 min at room temperature then kept on ice. For laser trap experiments, 3 µl of the bead suspension was mixed with 20 µl of BRB80, 1 mM DTT, 1 mM Mg^2+^ATP, 20 µM Taxol, 3 mg/ml glucose, 100 mg/ml glucose oxidase, 20 mg/ml catalase and the solution flushed into a flow chamber. A laser trap [76] was used to place the beads onto selected microtubules, and the trap then turned off to allow the bead to move freely. Because of the tendency of Klp6 to bind free tubulin, it was necessary to flush the flow cell prior to the experiment, in order to minimize the free tubulin concentration.

### Microtubule polymerisation assay

*S. pombe* or pig brain tubulin was diluted to 1.5 µM final concentration in BRB80, 1 mM DTT, 1 mM GMPCPP in a quartz cuvette. Microtubule polymerisation was assayed by 90° light scattering at 350 nm using a Cary Eclipse fluorometer (Varian) with a Peltier temperature controller at 25°C. After establishment of a baseline, desalted Klp6_440_His was added at different final concentrations. Samples were taken at t = 0 and t = 37 min for examination by dark field microscopy.

### Microtubule pelleting assay

100 µl aliquots of 350 nM of GMPCPP stabilised pig brain tubulin, Klp5_436_GST in K-PEM pH 6.9, 100 mM KCl, 0.1% (V/V) NP40, 1 mM DTT, 2 mM Mg.ATP were incubated at 25°C, centrifuged 88225 g, 10 min, 25°C, (TLA100 rotor, Optima TLX centrifuge, Beckman). 50 µl of supernatant was removed, the remainder discarded and the pellet resuspended in 100 µl of assay buffer (15 min on ice). Aliquots of pellet and supernatant were separated by SDS-PAGE (Invitrogen), stained (Sypro Red, Invitrogen) imaged (Molecular imager FX, BioRad) and quantified (Quantity one software, BioRad).

### Darkfield assays of microtubule stability

To create a sensitive assay for acceleration of depolymerisation, GMPCPP (Jena Bioscience GmbH) stabilised pig brain microtubules were diluted to 350 nM (Klp6_440_His bundling) or 100 nM (other experiments) in K-PEM pH 6.9, 100 mM KCl, 0.1% (v/v) NP40, 1 mM DTT, 2 mM Mg.ATP, 25°C, causing spontaneous depolymerisation. Kinesin was added, and time-point samples removed and mounted under an untreated or poly-L-Lysine coated coverslip and sealed with nail polish.

Coverslips (22×22 mm, No 1.5 Menzel-Gläser) and slides (1–1.2 mm Menzel-Gläser) were prepared by ultrasonic cleaning in 3% (v/v) Neutricon detergent (Decon Laboratories Ltd) using a 600 W ultrasonic bath (Ultrawave Ltd), followed by extensive rinsing and ultrasonic treatment in ultra pure water to create a hydrophilic surface. Then rinsed in 80% (v/v) ethanol and dried using a coverslip spinner (Technical Video Ltd). 40 µl of 0.1 mg/ml poly-L-lysine in H_2_O (Sigma >300, 000 mw) was added to the coverslip, and then excess poly-L-lysine removed using 3×150 µl H_2_O washes and a coverslip spinner before air-drying.

Microtubules were imaged using an Ixon DU-897E EM-CCD camera (Andor Technology PLC) and Metamorph software (Molecular Devices), E800 microscope, 1.4NA darkfield condenser, Plan Fluor 100×1.3NA iris objective, Green interference filter (Nikon) with 100 W mercury vapour lamp illumination via fibre optic light scrambler and cold mirror (Technical Video Ltd). Microtubule lengths were measured using Metamorph and analysed using Kaleidagraph (Synergy Software) and Prism software (Graphpad Software Inc).

### Microtubule dynamics assays

Microtubule dynamics assays [2] used flow cells of hydrophilic, ultrasonically cleaned 22×22 mm coverslips (No 1.5 Menzel-Gläser) and glass slides (1–1.2 mm Menzel-Gläser) separated by Parafilm spacers (Pechiney Plastic Packaging). Flow cells were flushed with *Echinus esculentus* sea-urchin sperm tail axoneme fragments [77], 5 chamber volumes of dynamics buffer (K-PEM pH 6.9, 50 mM K-Acetate, 1 mM MgSO_4_, 1 mM ATP, 1 mM GTP, 100 µg/ml BSA, 10 mM phosphocreatine, 50 µg/ml Creatine kinase), 5 volumes of dynamics buffer containing *S. pombe* tubulin and kinesin and sealed using VALAP (1∶1∶1 by weight Vaseline, lanolin and soft paraffin wax). Microtubules were imaged at 2 sec intervals by video enhanced DIC microscopy at 25°C using a video rate CCD camera (C3077, Argus 20 video processor, 2 frame averaging, Hamamatsu) and LG3 frame grabber card (Scion Corp) on a Mac G4 computer running NIH image software (US, National Institutes of Health, http://rsb.info.gov/nih-image/). Microtubule end positions were digitised manually (MT length measure custom Macro for NIH image). Periods of growth and shrinkage in plots of microtubule length against time were fitted by linear regression using Kaleidagraph (Synergy Software). Length changes <0.24 µm were classified as pauses. Catastrophe and rescue frequencies are total number of events divided by total growth or shrinkage time. Re-growth from an axoneme was not counted as a rescue.

### Live cell imaging

For live cell analysis, 1 ml of a log-phase culture of *S. pombe* in EMMG medium, 4 µM thiamine and appropriate amino acids was concentrated 100 times by centrifugation and a small drop mounted for microscopy as described [48]. Cells were observed using a Nikon Eclipse E800 fluorescence microscope equipped with an Andor iXon EMCDD camera (Andor technology), Nikon PlanApo ×100/1.40 NA oil objective lens and bandpass EGFP filter set (Chroma). Images were collected using MetaMorph software (Molecular Devices) and subsequently deconvolved by Autoquant software (MediaCybernetics).

### Measurement of microtubule dwell time at cell ends

For time-lapse series, 8 Z-sections (0.6 µm step size) were taken every 4 seconds and maximum intensity projections created using Metamorph Software. The dwell time at the end of the cell was defined by the interval between the first frame showing contact of the microtubule with the cell edge in the end zone and the first frame showing microtubule shrinkage. The cumulative frequency curves were fitted with a single exponential using Prism software.

## Supporting Information

Figure S1 **Purification and Characterisation of Full length Klp5 and Klp6 expressed in bacteria.** (A) Full length constructs. Klp5_FL_GST: Full-length Klp5 containing amino acids 1–883 fused to a C-terminal GST tag. Klp6_FL_His: Full length Klp6 containing amino acids 1–784 fused to a C-terminal His tag. The predicted molecular mass of the constructs (including tags) are 125 448 kDa and 88 738 kDa respectively. (B) Purification of Klp5_FL_GST/Klp6_FL_His co-expressed in bacteria. Colloidal Coomassie blue stained SDS-PAGE separations of bacterial lysate; post His-tag affinity purification on a Ni resin column, and sequential GST-tag affinity purification of the His-tag purified proteins showing the fractions eluted by 10 mM Glutathione from a GST-tag affinity column. The molar ratio of Klp6_FL_His ∶ Klp5_FL_GST was 3.2∶1 as determined by densitometry of a Colloidal Coomassie blue stained SDS-PAGE separation of the final sequentially purified fraction. (C) Klp6_FL_His expressed in bacteria, purified by Nickel affinity chromatography, separated by SDS-PAGE and visualised by Colloidal Coomassie blue staining.
(TIF)

Figure S2 **Velocity Distribution of** ***S. pombe*** **microtubules in a Klp6_440_His Microtubule sliding motility assay.** The mean velocities of microtubules sliding on a surface of Klp6_440_His in presence (16±5 nm s^−1^ (54)) or absence (23±12 nm s^−1^, (77)) of 75 mM KCl were not significantly different. Velocities are mean ± SD (n).
(TIF)

Figure S3 **Microtubule Depolymerisation assay of Klp6_440_His and MCAK.** Microtubule depolymerisation was assayed by 90° light scattering at 350 nm, 25°C. The lightscattering of the buffer solution containing BRB80 (80 mM PIPES, 1 mM EDTA, 1 mM MgCl_2_, pH 6.9), 1 mM ATP before addition of proteins is indicated by a dashed black line. Addition of 1 µM of GMPCPP stabilised pig brain tubulin microtubules (indicated by arrow) caused an increase in lightscattering above background. Addition of CaCl_2_ alone (green) depolymerises the microtubules and decreases light scattering back to the buffer only level. Upon addition (indicated by arrow) of MCAK (black trace) or Klp6_440_His (red trace) to the microtubule solution an immediate further increase in light scattering was observed which may correspond to kinesin binding to the microtubules. In the presence of MCAK, the light scattering decreases to the level corresponding to buffer alone before addition of microtubules. Addition of CaCl_2_ (indicated by arrow) causes no further decrease in light scattering. In the presence of Klp6_440_His, there was a decrease in light scattering to a level higher than with microtubules alone. This level remains constant until the addition of CaCl_2_ which causes a slower rate of decrease in light scattering than the one observed for microtubules alone (green trace). Samples of the Klp6_440_His assay examined by VE-DIC microscopy contain microtubules before adding CaCl_2_ (A) and fewer, mostly bundled, microtubules after adding CaCl_2_ (B). Scale bar: 5 µm.
(TIF)

Figure S4 **Depolymerase pelleting assay with GMPCPP stabilised pig brain microtubules.** A pelleting assay was used to determine the effect of Klp5_436_GST on GMPCPP stabilised pig brain tubulin microtubules. 350 nM of GMPCPP pig brain microtubules were incubated with increasing concentrations of Klp5_436_GST at 25°C for up to 60 min before pelleting the microtubules. The plot of the percentage of total tubulin in the supernatant shows that under the assay conditions the GMPCPP stabilised pig brain tubulin microtubules are spontaneously depolymerising. Addition of Klp5_436_GST causes this rate of depolymerisation to decrease suggesting that rather than accelerating depolymerisation under these conditions, Klp5_430_GST appears to stabilise the microtubules. Klp6_440_His and tubulin have similar migration on SDS-PAGE. Therefore, in pelleting assays although we could exclude large effects of Klp6_440_His or Klp6_440_His/Klp5_436_GST on MT depolymerisation we could not exclude effects that are more modest.
(TIF)

Figure S5 **Depolymerisation assay.** Darkfield images showing (A) 100 nM of GMPCPP stabilised pig brain tubulin microtubules incubated at 25°C for 0.5 and 47 minutes, (B) 100 nM microtubules plus 50 nM MCAK for 0.5 and 45 minutes. (C) 100 nM of microtubules for 0.5, 40 and 65 minutes, (D) 100 nM microtubules plus 50 nM Klp6_415_His for 0.5, 35 and 70 minutes and (E) 100 nM of microtubules plus 100 nM Klp6_415_His for 0.5, 40 and 65 minutes. A significant decrease in microtubule number was observed on incubation with MCAK (B) compared to microtubules alone (A), however no significant difference was observed, even on prolonged incubation in microtubules with (D, E) and without Klp6_415_His (C). No bundling of microtubules was observed with Klp6_415_His.
(TIF)

Figure S6 **His-Klp6_FL_ effect upon dynamic** ***S. pombe*** **tubulin microtubules.** VE-DIC images of (A) 0 nM, (B) 164 nM and (C) 2,066 nM of His-Klp6_FL_ kinesin heads in MT dynamics assays with 4.4 µM *S. pombe* tubulin at 25°C. Scale bar 5 µm. At 2,066 nM of His-Klp6_FL_ (C) many spontaneously nucleated MTs are observed, whilst at lower concentrations MTs are nucleated from the axoneme fragments (A, B). Sliding of MTs over the surface was also observed in (C) showing that the His-Klp6_FL_ construct has motor activity as well as MT stimulated ATPase. The high level of spontaneously nucleated MTs meant that the 2,066 nM sample (C) MT dynamics could not be analysed.
(TIF)

Figure S7 **Co-expressed Klp5_436_GST/Klp6_440_His effect upon dynamic** ***S. pombe*** **tubulin microtubules.** VE-DIC of 4 µM of *S. pombe* GTP tubulin with (A) 0 nM, (B) 372 nM, (C) 3720 nM of KLP5_436_GST/KLP6_440_His and (D) 42 nM of MT affinity purified KLP5_436_GST/KLP6_440_His in microtubule dynamics assays at 25°C. Scale bar 5 µm. In 42 nM of affinity purified KLP5_436_GST/KLP6_440_His (D) some rare spontaneous MT formation and motility of MTs on the surface was observed. 372 nM of KLP5_436_GST/KLP6_440_His (B) causes more spontaneous MT formation, which increased to frequent spontaneous MT formation together with bundling and motility on the surface in 3720 nM (C).
(TIF)

Figure S8 **Klp5_436_GST effect upon dynamic** ***S. pombe*** **tubulin microtubules.** VE-DIC of 3.5 µM *S. pombe* GTP tubulin with (A) 0 nM, (B) 85 nM, (C) 170 nM and (D) 2,960 nM Klp5_436_GST in microtubule dynamics assays at 25°C. Scale bar 5 µm. The 2,960 nM Klp5_436_GST sample (D) caused extensive spontaneous nucleation, bundling and motility within the assay. At lower concentrations these effects were reduced, but not completely absent (B, C).
(TIF)

Figure S9 **Klp5 and Klp6 cause microtubule bundling.** Darkfield images showing (A) 100 nM GMPCPP stabilised pig brain tubulin microtubules incubated at 25°C for 0.5, 37 and 85 minutes; (B) 100 nM microtubules plus 21 nM Klp5_436_GST for 0.5 and 42 minutes; (C) 100 nM microtubules plus Klp5_436_GST/Klp6_440_His (50 nM Klp5 heads/6.9 nM Klp6 heads, determined by gel staining of the desalted sample) for 0.5 minutes; (D) 100 nM microtubules plus 50 nM Klp5_436_ (GST removed) for 0.5 and 30 minutes and (E) 350 nM microtubules and 100 nM Klp6_440_His for 0.5 minutes. A, B, C and D are shown to same scale with scale bar (in D) equivalent to 5 µm. Scale bar in E is also equivalent to 5 µm. Pig brain tubulin GMPCPP microtubules were diluted to 350 or 100 nM concentration. In these conditions the microtubules spontaneously depolymerise so any effects of the kinesins upon this rate of depolymerisation should be easily detected. Klp5 and Klp6 were added to the microtubules in solution then aliquots removed and examined by darkfield microscopy. We found that the constructs Klp5_436_GST (B), Klp6_440_His (E) or Klp5_436_GST/Klp6_440_His (C) all caused bundling of the preformed microtubules under conditions where microtubules alone did not bundle. Removal of the GST tag from Klp5_436_ (D) or reduced ionic strength by omitting 100 mM KCl from the buffer or omitting ATP from buffer (data not shown) did not prevent bundling by this construct. Only Klp6_415_His, which omits the predicted dimerisation domain, did not cause any significant bundling even upon prolonged incubation (supplementary fig S5). These results suggest that the bundling activity, at least for Klp6 depends upon crosslinking via both of the kinesin heads in multiheaded constructs. It also supports Klp6 forming functional dimers or multimers.
(TIF)

Figure S10 **Klp6_440_His ATPase assay for competition between microtubule and tubulin heterodimer binding.** 100 nM Klp6_440_His and 73 nM of Taxol stabilised pig brain microtubules were added to an ATPase assay where the decrease in absorbance at OD_340_ is directly linked to the ATPase activity in the assay. After incubation for 75 seconds pig brain tubulin heterodimers were added to 1 µM final concentration, with a break in monitoring during mixing. A new steady state of ATPase activity is then established rapidly. The decrease in slope of the new line (by 66% in the example shown) compared to the initial conditions (indicated by the dashed line) shows the inhibitory effect of the tubulin heterodimers in competition with the microtubules for activation of Klp_440_His ATPase. Typically the microtubule activated ATPase is inhibited within 20 sec following addition of an excess of pig brain tubulin and a new steady state of lower ATPase activity (due to the very low ATPase activation by the tubulin heterodimer) is established within ∼100 s.
(TIF)

Movie S1 **Microtubule sliding assay of Klp5.** Time-lapse movie (1 frame /40 s) of Klp5 driven motility of Rhodamine tagged pig brain microtubules imaged by fluorescence microscopy at 25°C. The microtubules are polarity marked by more intensely stained microtubule seeds at their minus end. Pixel size is 0.130 µm.
(MOV)

Movie S2 **Microtubule sliding assay of Klp6.** Time-lapse movie (1 frame /40 s) of Klp6 driven motility of Rhodamine tagged pig brain microtubules imaged by fluorescence microscopy at 25°C. The microtubules are polarity marked by more intensely stained microtubule seeds at their minus end. Pixel size is 0.130 µm.
(MOV)

Movie S3 **Wild type** ***S. pombe*** **.** Time-lapse movie (1 frame /4 s) of a wild type strain of *S. pombe*, containing a GFP α2 tubulin fusion, imaged by fluorescence microscopy at 25°C. Pixel size is 0.107 µm.
(MOV)

Movie S4 ***S. pombe*** **with** ***klp5*** **deletion.** Time-lapse movie (1 frame /4 s) of a Δ*klp5* strain of *S. pombe*, containing a GFP α2 tubulin fusion, imaged by fluorescence microscopy at 25°C. Pixel size is 0.107 µm.
(MOV)

Movie S5 ***S. pombe*** **with** ***klp6*** **deletion.** Time-lapse movie (1 frame /4 s) of a Δ*klp6* strain of *S. pombe*, containing a GFP α2 tubulin fusion, imaged by fluorescence microscopy at 25°C. Pixel size is 0.107 µm.
(MOV)

Movie S6 ***S. pombe*** **with** ***klp6*** **deletion.** Time-lapse movie (1 frame /4 s) of a Δ*klp6* strain of *S. pombe*, containing a GFP α2 tubulin fusion, imaged by fluorescence microscopy at 25°C. Pixel size is 0.107 µm. Movie shows the shrinkage of a microtubule towards the cell end-zone rather than towards the cell centre as observed in wild type cells.
(MOV)

Table S1 **Microtubule Activated ATPase activity of full length Klp6_FL_His.** Microtubules were assembled from either pig brain tubulin and stabilised with Taxol or *S. pombe* tubulin stabilised with GMPCPP. Microtubule or tubulin heterodimer stimulated ATPase activities of Klp6_FL_His were determined in linked assays at 25°C. Non-linear fits of plots of ATPase activity against tubulin concentration were used to determine Vmax and Km values. No tubulin stimulated ATPase activity was detected and Vmax and Km values for tubulin stimulation could not be determined.
(DOC)

Table S2 **Microtubule Velocities reported for kinesins-8.** Published values for microtubule sliding velocities on surfaces of Kinesin-8 motors and for velocity of single molecules of Kinesin-8 moving on microtubules.
(DOC)

Table S3 **Velocity and dwell times of Klp6_440_His coated beads on pig brain tubulin microtubules.** An optical trap was used to position Klp6_440_His coated beads on Taxol stabilised microtubules assembled from pig brain tubulin (figure 2). The velocity of bead translocation along the microtubule and subsequent dwell time at the microtubule tip was measured for 6 beads. The mean dwell time was 42±10 s (6) and velocity 47±7 nm/s (5) (mean ± SEM (n)). The average velocity is slower than is observed in motility assays (87±18 nm/s (1085)), which may be caused by different buffers or surface densities of Klp6_440_His in the two assays. Bead movement was processive at high motor concentrations. At low motor densities beads failed to attach and move along microtubules.
(DOC)

Table S4 **Effect of Klp_436_GST upon GMPCPP stabilised pig brain Microtubule depolymerisation rates.** Microtubule bundling activity of Klp5 and klp6 (see supplementary figure S9) prevented detailed analysis of their effect upon microtubule stability in solution by microscopic methods, apart from noting that even in incubations lasting up to 70 min numerous bundled microtubules were still present at the end of the incubation period. To test if this bundling activity might be masking a depolymerase activity the effect of klp5_436_GST upon individual microtubules was tested by first binding single microtubules to a poly-lysine coated surface before addition of the kinesin and imaging by dark field microscopy. Time-lapse movies were recorded then analysed using the kymograph function of Metamorph software. Spontaneous depolymerisation of the GMPCPP microtubules at 0.40 nm s^−1^ was still observed. Although addition of Klp5_436_GST caused no bundling, depolymerisation was not significantly enhanced suggesting that bundling was not masking a depolymerase activity.
(DOC)

Table S5 **His-Klp6_FL_ effect upon** ***S. pombe*** **GTP microtubule fast end dynamics.** Effect of His-Klp6_FL_ on fast end microtubule dynamics in assays at 25°C containing 4.4 µM *S. pombe* GTP tubulin with microtubules nucleated by axoneme fragments.
(DOC)

Table S6 **His-Klp6_FL_ effect upon** ***S. pombe*** **GTP microtubule slow end dynamics.** Effect of His-Klp6_FL_ on slow end microtubule dynamics in assays at 25°C containing 4.4 µM *S. pombe* GTP tubulin with microtubules nucleated by axoneme fragments.
(DOC)

Table S7 **Klp5_436_GST/Klp6_440_His effect upon** ***S. pombe*** **GTP microtubule fast end dynamics.** Effect of KLP5_436_GST/KLP6_440_His on fast end microtubule dynamics in assays at 25°C containing 4.0 µM *S. pombe* GTP tubulin with microtubules nucleated by axoneme fragments.
(DOC)

Table S8 **Klp5_436_GST/Klp6_440_His effect upon** ***S. pombe*** **GTP microtubule slow end dynamics.** Effect of KLP5_436_GST/KLP6_440_His on slow end microtubule dynamics in assays at 25°C containing 4.0 µM *S. pombe* GTP tubulin with microtubules nucleated by axoneme fragments.
(DOC)

Table S9 **Klp5_436_GST effect upon** ***S. pombe*** **GTP microtubule fast end dynamics.** Effect of Klp5_436_GST on fast end microtubule dynamics in assays at 25°C containing 3.5 µM *S. pombe* GTP tubulin with microtubules nucleated by axoneme fragments.
(DOC)

Table S10 **Klp5_436_GST effect upon** ***S. pombe*** **GTP microtubule slow end dynamics.** Effect of Klp5_436_GST on slow end microtubule dynamics in assays at 25°C containing 3.5 µM *S. pombe* GTP tubulin with microtubules nucleated by axoneme fragments.
(DOC)

Table S11 **Oligonucleotides used in creating Klp5 and 6 constructs.** Oligonucleotides used in PCR reactions to create the Klp5 and 6 protein expression vectors.
(DOC)
