# Supplementary figures and images for: S. pombe Kinesins-8 Promote Both Nucleation and Catastrophe of Microtubules
Source: PLoS One. 2012 Feb 20;7(2):e30738. doi: 10.1371/journal.pone.0030738 (PMC3282699; doi:10.1371/journal.pone.0030738)

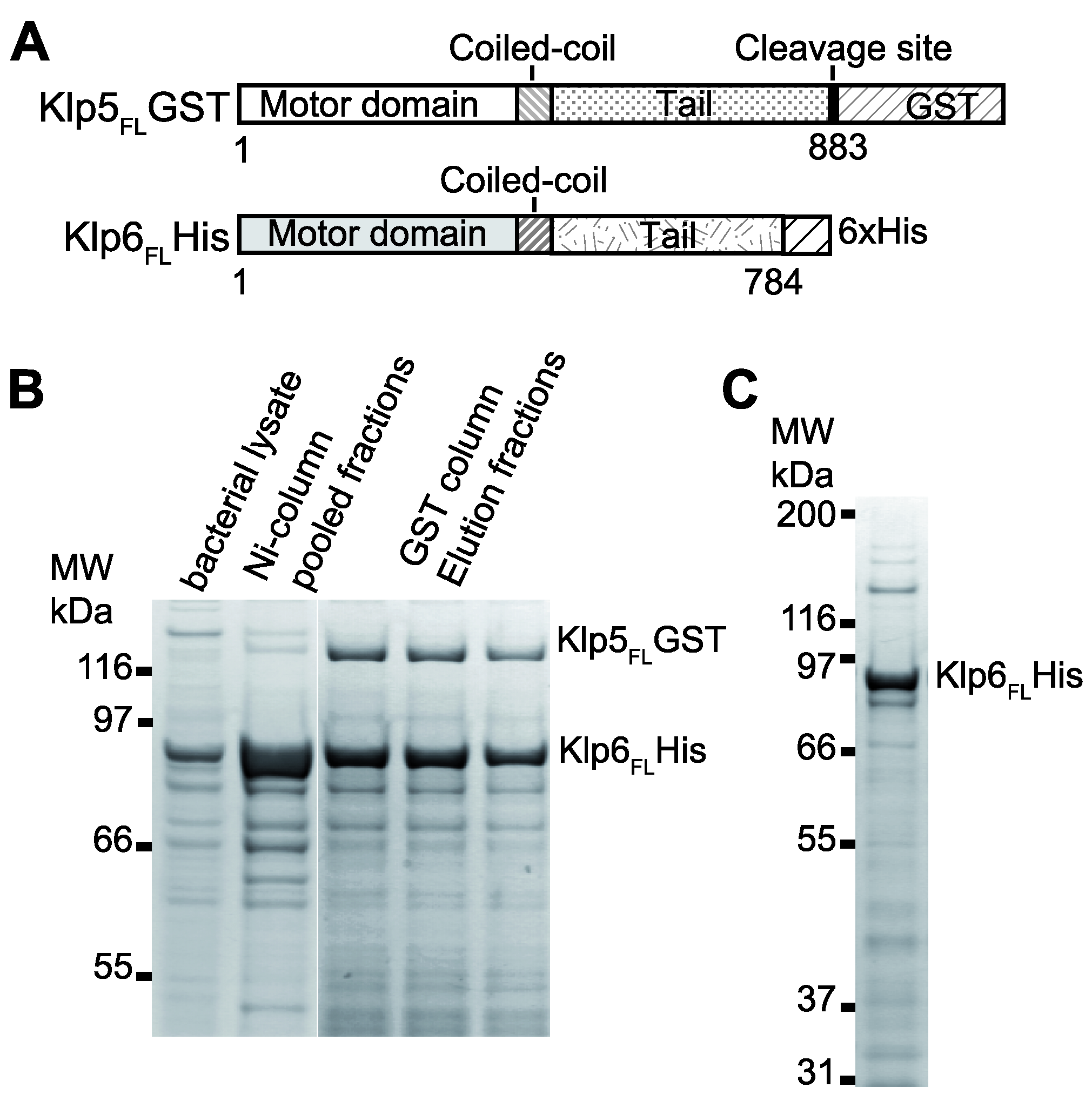

Supplement: Figure S1 — Purification and Characterisation of Full length Klp5 and Klp6 expressed in bacteria. (A) Full length constructs. Klp5FLGST: Full-length Klp5 containing amino acids 1–883 fused to a C-terminal GST tag. Klp6FLHis: Full length Klp6 containing amino acids 1–784 fused to a C-terminal His tag. The predicted molecular mass of the constructs (including tags) are 125 448 kDa and 88 738 kDa respectively. (B) Purification of Klp5FLGST/Klp6FLHis co-expressed in bacteria. Colloidal Coomassie blue stained SDS-PAGE separations of bacterial lysate; post His-tag affinity purification on a Ni resin column, and sequential GST-tag affinity purification of the His-tag purified proteins showing the fractions eluted by 10 mM Glutathione from a GST-tag affinity column. The molar ratio of Klp6FLHis ∶ Klp5FLGST was 3.2∶1 as determined by densitometry of a Colloidal Coomassie blue stained SDS-PAGE separation of the final sequentially purified fraction. (C) Klp6FLHis expressed in bacteria, purified by Nickel affinity chromatography, separated by SDS-PAGE and visualised by Colloidal Coomassie blue staining. (TIF) [file pone.0030738.s001.tif]

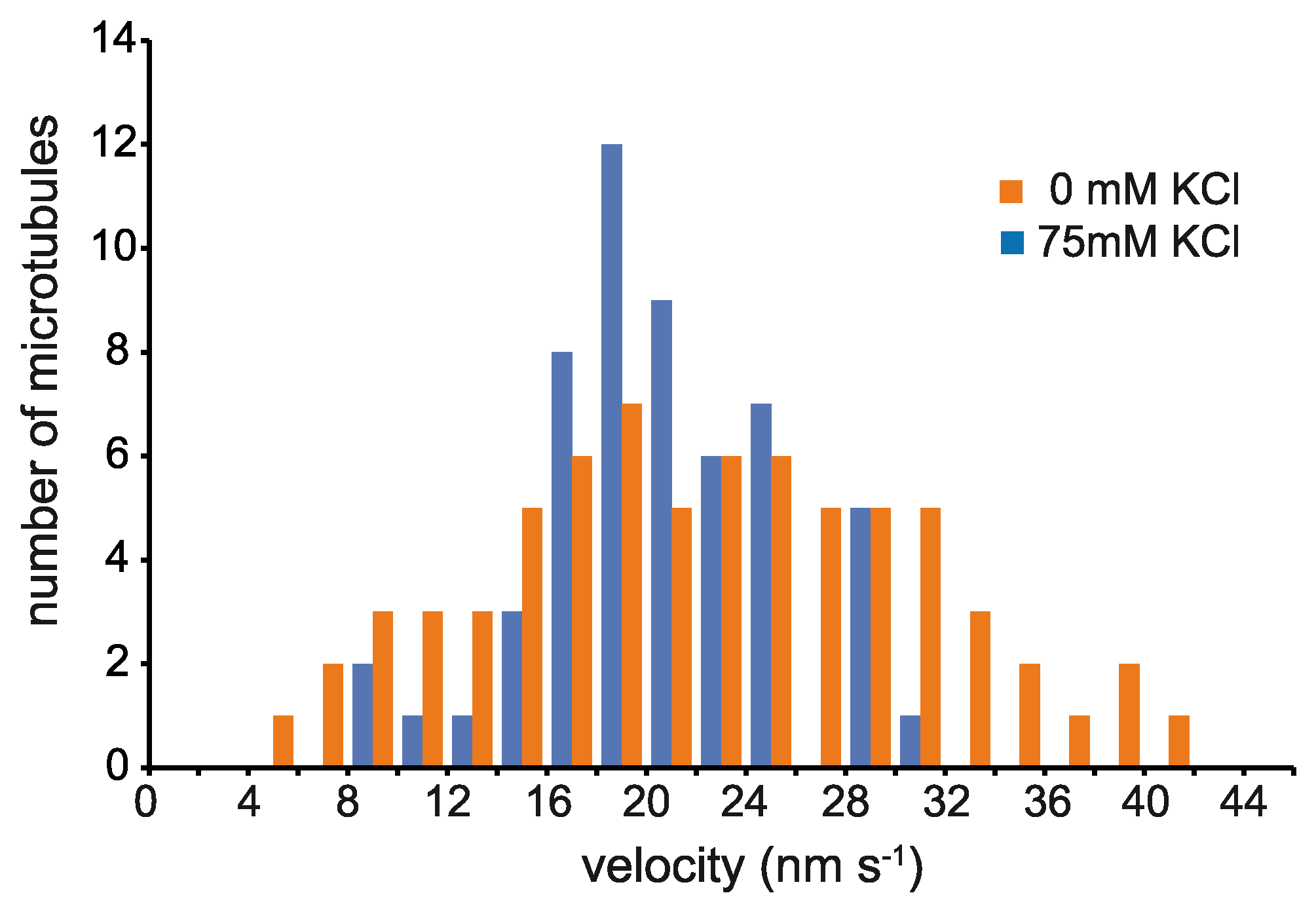

Supplement: Figure S2 — Velocity Distribution of S. pombe microtubules in a Klp6440His Microtubule sliding motility assay. The mean velocities of microtubules sliding on a surface of Klp6440His in presence (16±5 nm s−1 (54)) or absence (23±12 nm s−1, (77)) of 75 mM KCl were not significantly different. Velocities are mean ± SD (n). (TIF) [file pone.0030738.s002.tif]

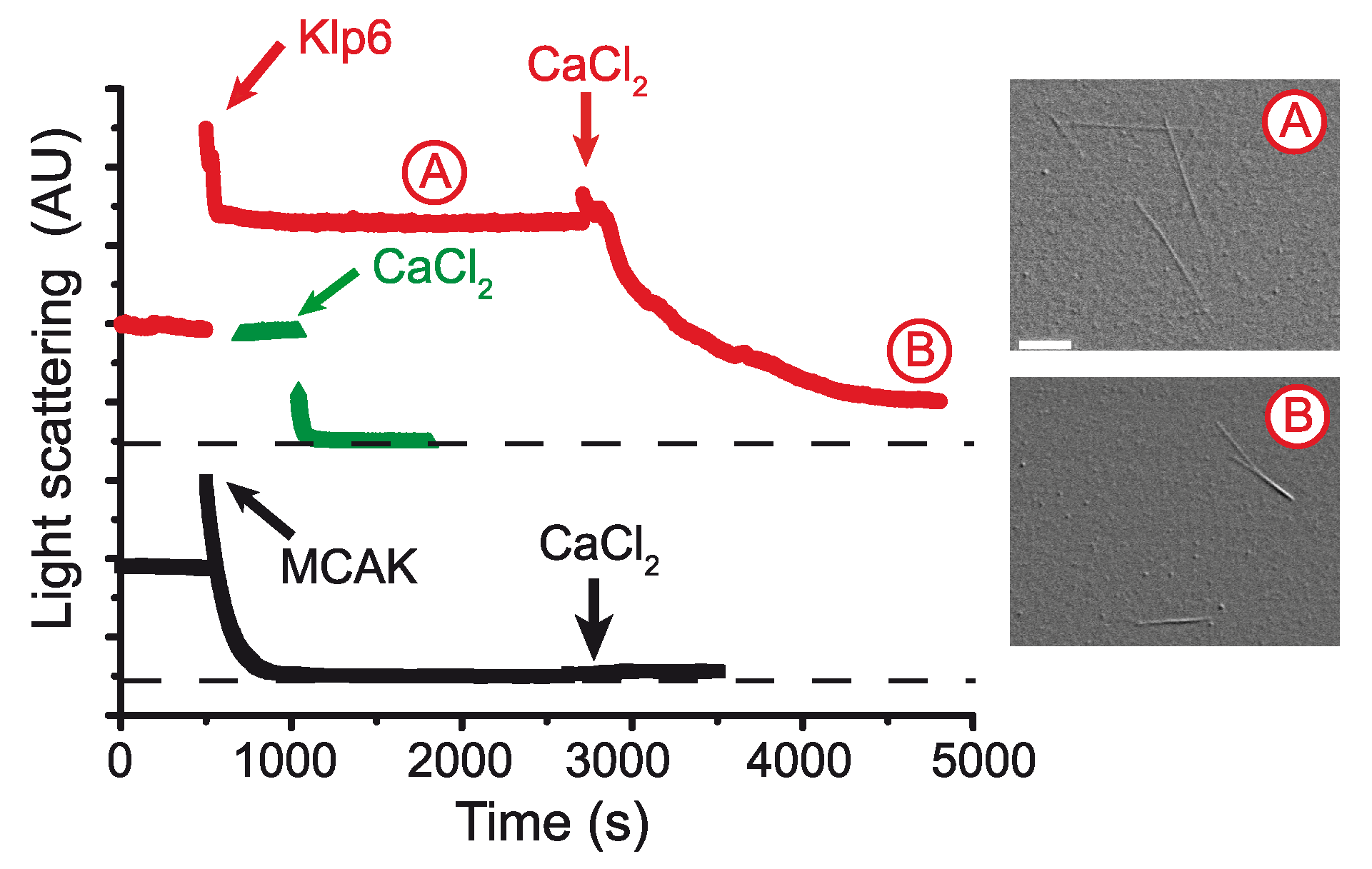

Supplement: Figure S3 — Microtubule Depolymerisation assay of Klp6440His and MCAK. Microtubule depolymerisation was assayed by 90° light scattering at 350 nm, 25°C. The lightscattering of the buffer solution containing BRB80 (80 mM PIPES, 1 mM EDTA, 1 mM MgCl2, pH 6.9), 1 mM ATP before addition of proteins is indicated by a dashed black line. Addition of 1 µM of GMPCPP stabilised pig brain tubulin microtubules (indicated by arrow) caused an increase in lightscattering above background. Addition of CaCl2 alone (green) depolymerises the microtubules and decreases light scattering back to the buffer only level. Upon addition (indicated by arrow) of MCAK (black trace) or Klp6440His (red trace) to the microtubule solution an immediate further increase in light scattering was observed which may correspond to kinesin binding to the microtubules. In the presence of MCAK, the light scattering decreases to the level corresponding to buffer alone before addition of microtubules. Addition of CaCl2 (indicated by arrow) causes no further decrease in light scattering. In the presence of Klp6440His, there was a decrease in light scattering to a level higher than with microtubules alone. This level remains constant until the addition of CaCl2 which causes a slower rate of decrease in light scattering than the one observed for microtubules alone (green trace). Samples of the Klp6440His assay examined by VE-DIC microscopy contain microtubules before adding CaCl2 (A) and fewer, mostly bundled, microtubules after adding CaCl2 (B). Scale bar: 5 µm. (TIF) [file pone.0030738.s003.tif]

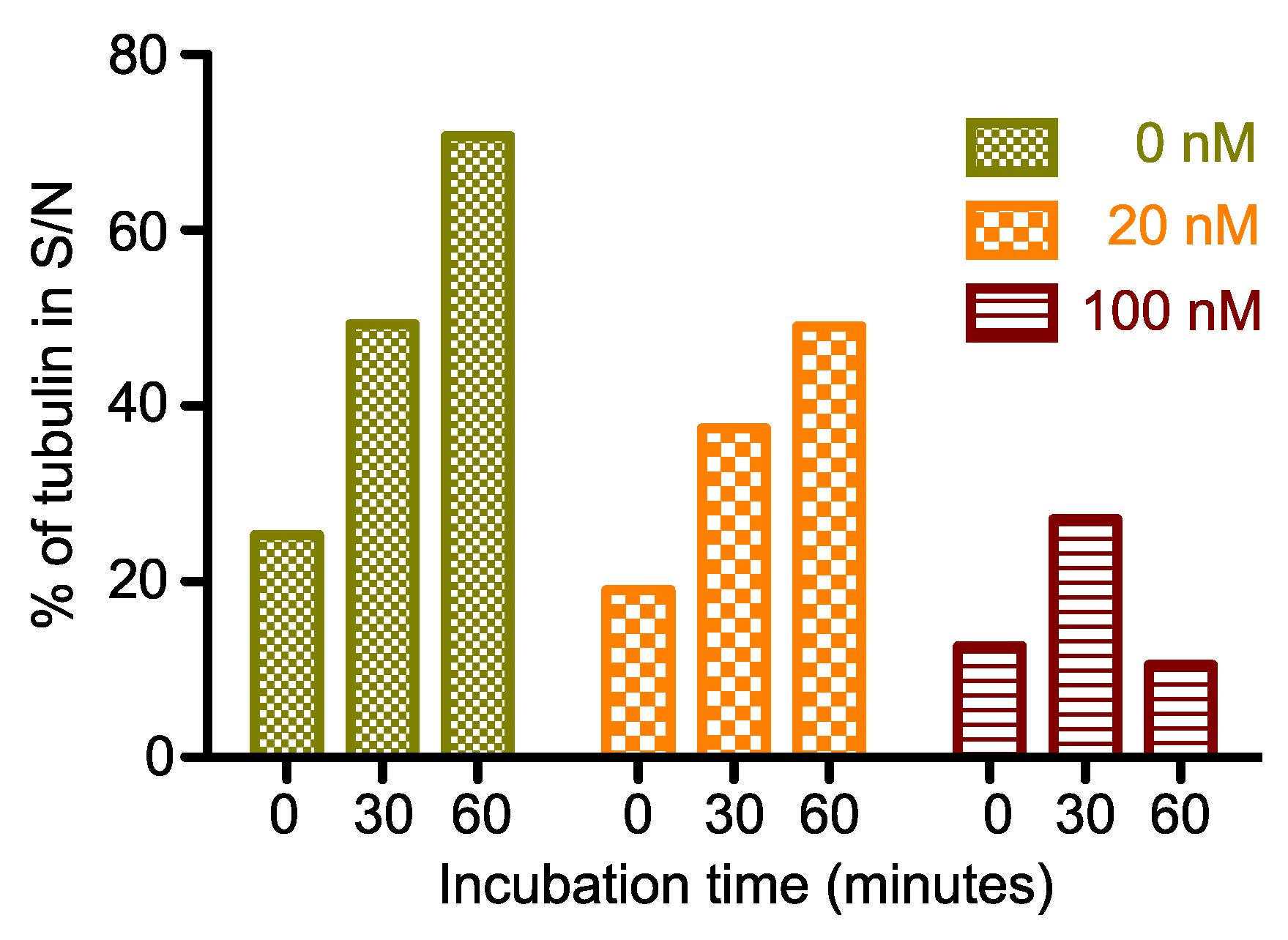

Supplement: Figure S4 — Depolymerase pelleting assay with GMPCPP stabilised pig brain microtubules. A pelleting assay was used to determine the effect of Klp5436GST on GMPCPP stabilised pig brain tubulin microtubules. 350 nM of GMPCPP pig brain microtubules were incubated with increasing concentrations of Klp5436GST at 25°C for up to 60 min before pelleting the microtubules. The plot of the percentage of total tubulin in the supernatant shows that under the assay conditions the GMPCPP stabilised pig brain tubulin microtubules are spontaneously depolymerising. Addition of Klp5436GST causes this rate of depolymerisation to decrease suggesting that rather than accelerating depolymerisation under these conditions, Klp5430GST appears to stabilise the microtubules. Klp6440His and tubulin have similar migration on SDS-PAGE. Therefore, in pelleting assays although we could exclude large effects of Klp6440His or Klp6440His/Klp5436GST on MT depolymerisation we could not exclude effects that are more modest. (TIF) [file pone.0030738.s004.tif]

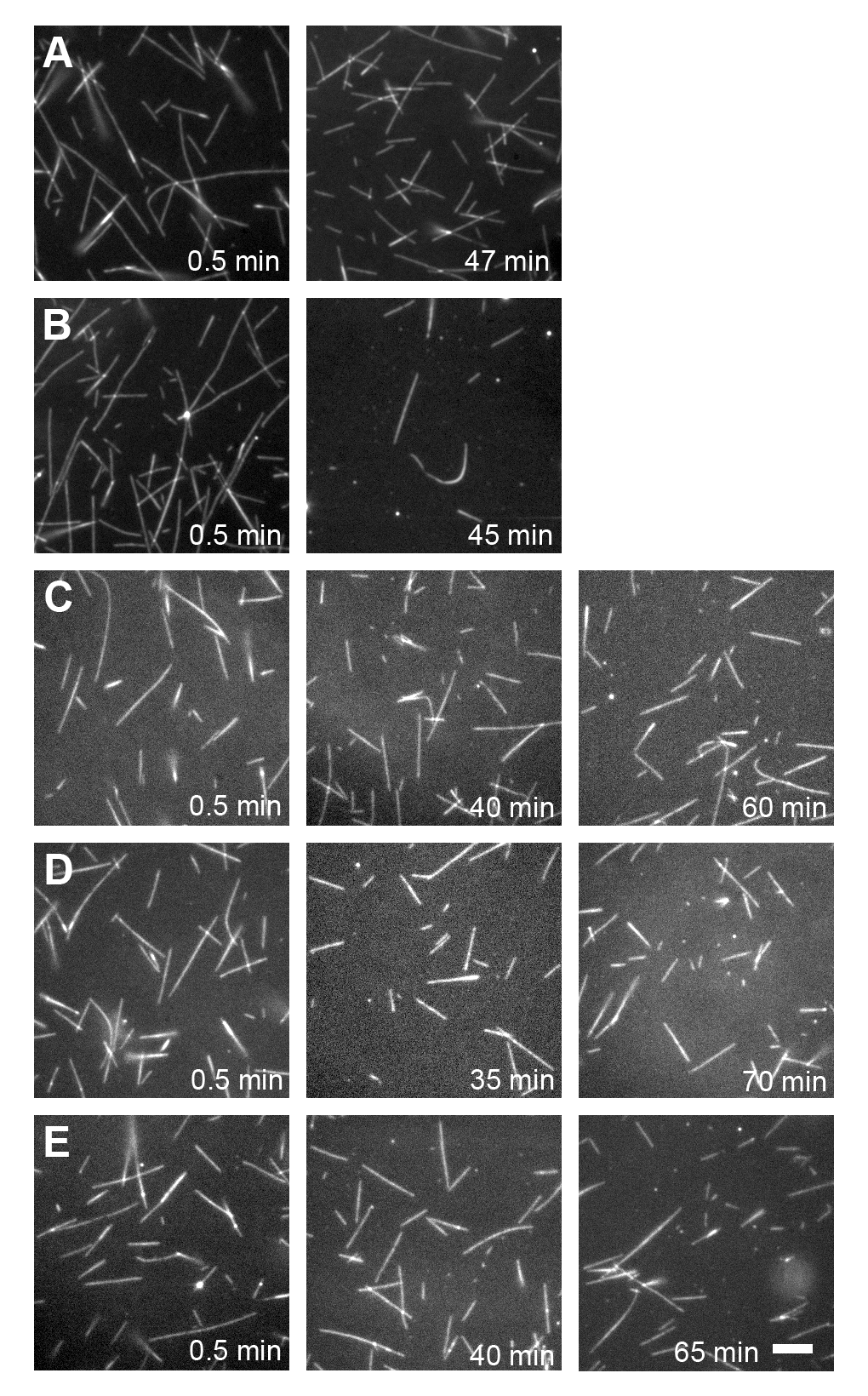

Supplement: Figure S5 — Depolymerisation assay. Darkfield images showing (A) 100 nM of GMPCPP stabilised pig brain tubulin microtubules incubated at 25°C for 0.5 and 47 minutes, (B) 100 nM microtubules plus 50 nM MCAK for 0.5 and 45 minutes. (C) 100 nM of microtubules for 0.5, 40 and 65 minutes, (D) 100 nM microtubules plus 50 nM Klp6415His for 0.5, 35 and 70 minutes and (E) 100 nM of microtubules plus 100 nM Klp6415His for 0.5, 40 and 65 minutes. A significant decrease in microtubule number was observed on incubation with MCAK (B) compared to microtubules alone (A), however no significant difference was observed, even on prolonged incubation in microtubules with (D, E) and without Klp6415His (C). No bundling of microtubules was observed with Klp6415His. (TIF) [file pone.0030738.s005.tif]

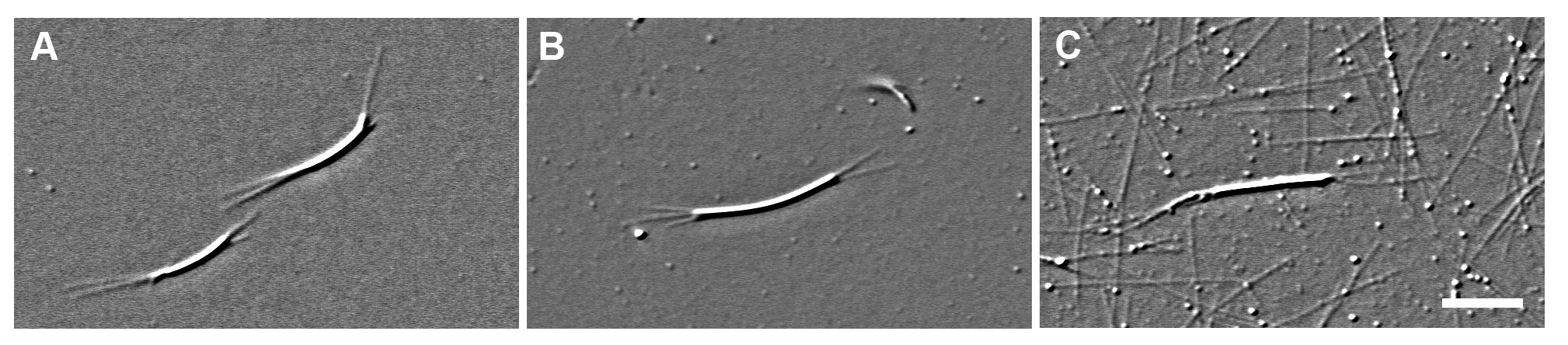

Supplement: Figure S6 — His-Klp6FL effect upon dynamic S. pombe tubulin microtubules. VE-DIC images of (A) 0 nM, (B) 164 nM and (C) 2,066 nM of His-Klp6FL kinesin heads in MT dynamics assays with 4.4 µM S. pombe tubulin at 25°C. Scale bar 5 µm. At 2,066 nM of His-Klp6FL (C) many spontaneously nucleated MTs are observed, whilst at lower concentrations MTs are nucleated from the axoneme fragments (A, B). Sliding of MTs over the surface was also observed in (C) showing that the His-Klp6FL construct has motor activity as well as MT stimulated ATPase. The high level of spontaneously nucleated MTs meant that the 2,066 nM sample (C) MT dynamics could not be analysed. (TIF) [file pone.0030738.s006.tif]

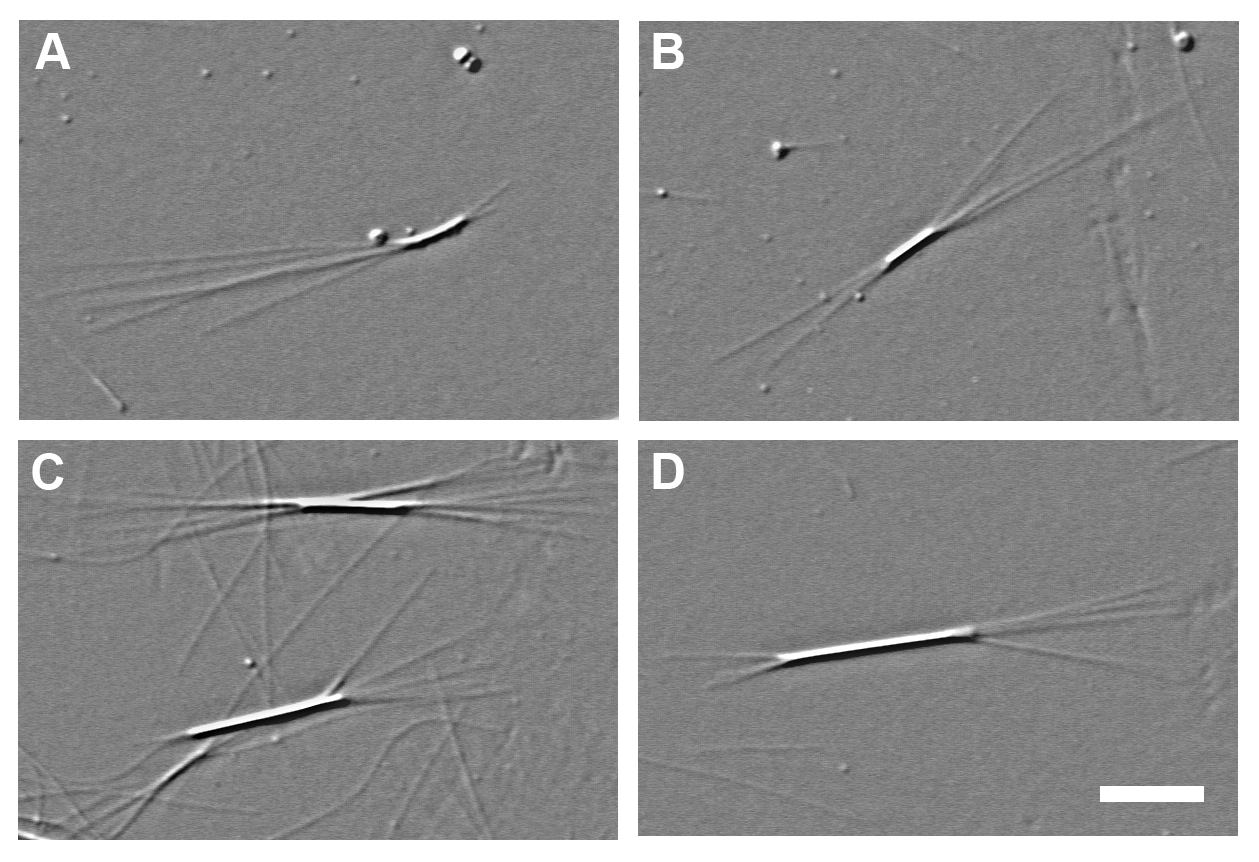

Supplement: Figure S7 — Co-expressed Klp5436GST/Klp6440His effect upon dynamic S. pombe tubulin microtubules. VE-DIC of 4 µM of S. pombe GTP tubulin with (A) 0 nM, (B) 372 nM, (C) 3720 nM of KLP5436GST/KLP6440His and (D) 42 nM of MT affinity purified KLP5436GST/KLP6440His in microtubule dynamics assays at 25°C. Scale bar 5 µm. In 42 nM of affinity purified KLP5436GST/KLP6440His (D) some rare spontaneous MT formation and motility of MTs on the surface was observed. 372 nM of KLP5436GST/KLP6440His (B) causes more spontaneous MT formation, which increased to frequent spontaneous MT formation together with bundling and motility on the surface in 3720 nM (C). (TIF) [file pone.0030738.s007.tif]

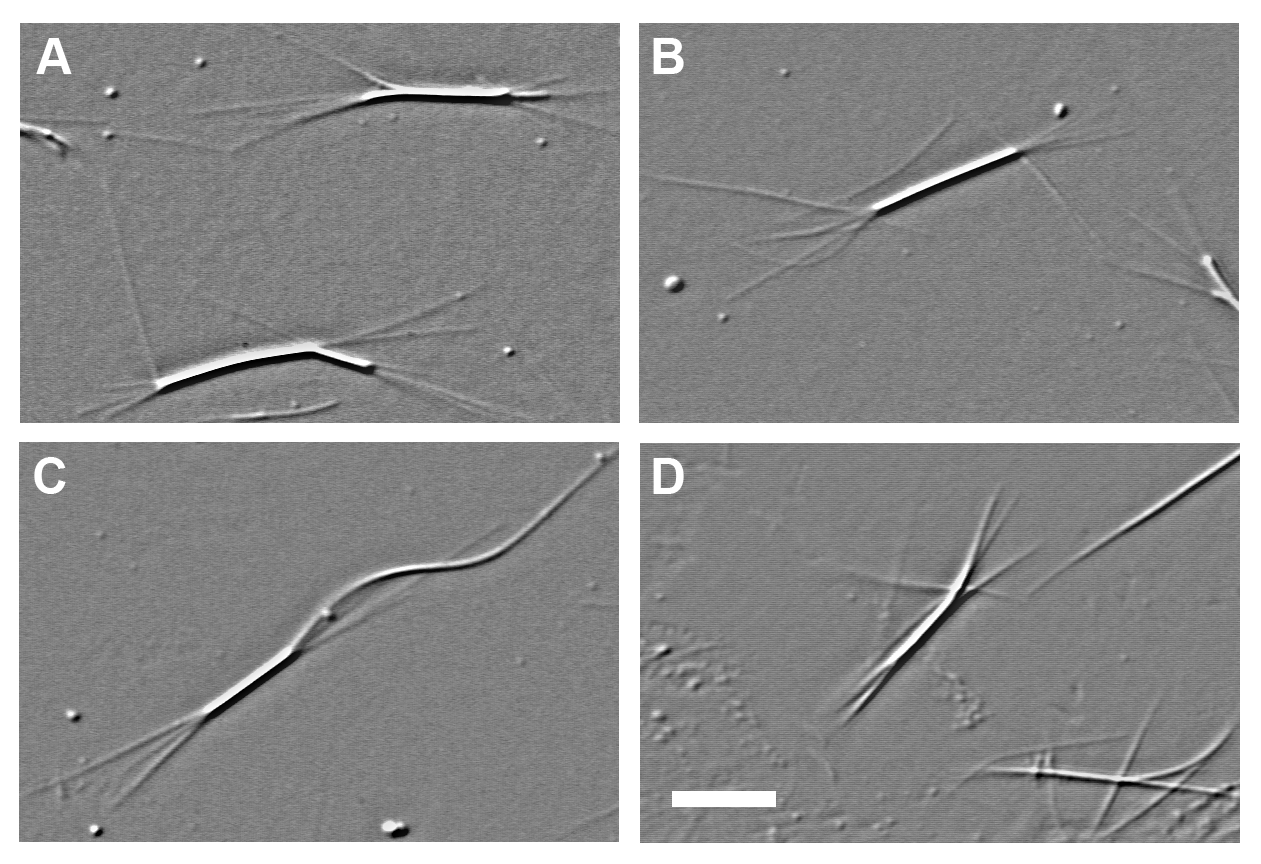

Supplement: Figure S8 — Klp5436GST effect upon dynamic S. pombe tubulin microtubules. VE-DIC of 3.5 µM S. pombe GTP tubulin with (A) 0 nM, (B) 85 nM, (C) 170 nM and (D) 2,960 nM Klp5436GST in microtubule dynamics assays at 25°C. Scale bar 5 µm. The 2,960 nM Klp5436GST sample (D) caused extensive spontaneous nucleation, bundling and motility within the assay. At lower concentrations these effects were reduced, but not completely absent (B, C). (TIF) [file pone.0030738.s008.tif]

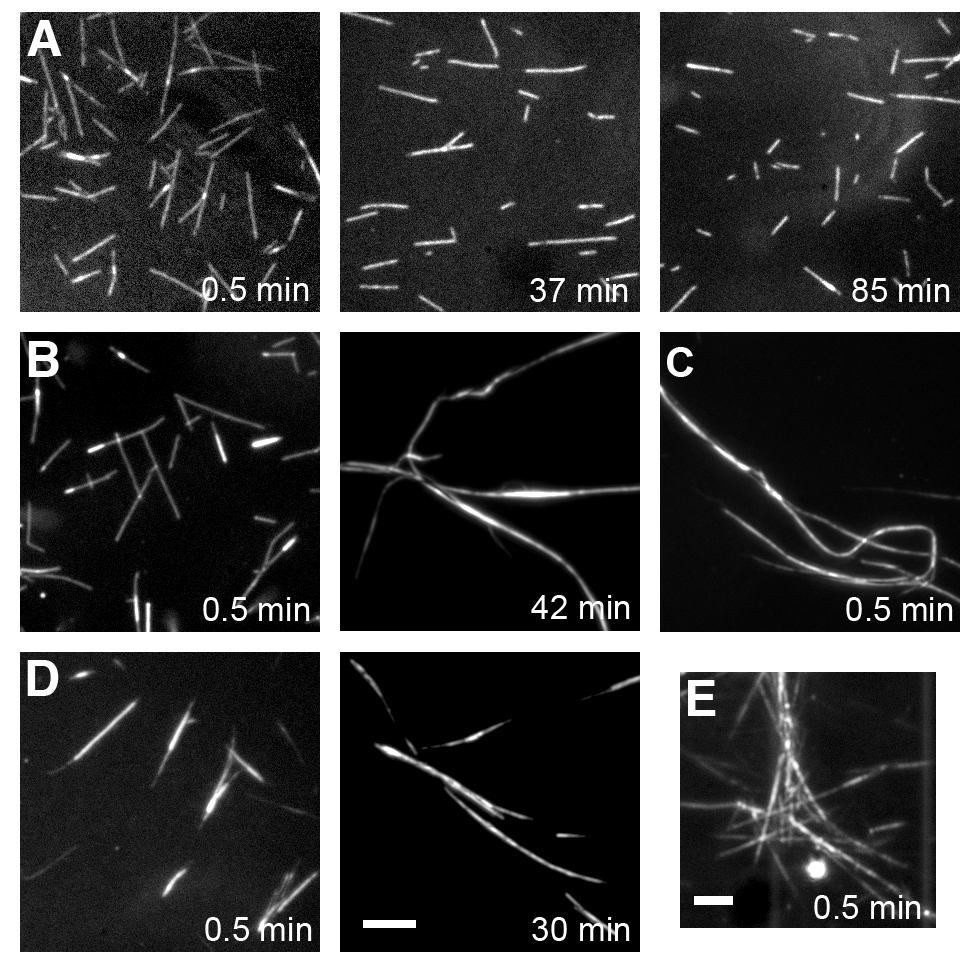

Supplement: Figure S9 — Klp5 and Klp6 cause microtubule bundling. Darkfield images showing (A) 100 nM GMPCPP stabilised pig brain tubulin microtubules incubated at 25°C for 0.5, 37 and 85 minutes; (B) 100 nM microtubules plus 21 nM Klp5436GST for 0.5 and 42 minutes; (C) 100 nM microtubules plus Klp5436GST/Klp6440His (50 nM Klp5 heads/6.9 nM Klp6 heads, determined by gel staining of the desalted sample) for 0.5 minutes; (D) 100 nM microtubules plus 50 nM Klp5436 (GST removed) for 0.5 and 30 minutes and (E) 350 nM microtubules and 100 nM Klp6440His for 0.5 minutes. A, B, C and D are shown to same scale with scale bar (in D) equivalent to 5 µm. Scale bar in E is also equivalent to 5 µm. Pig brain tubulin GMPCPP microtubules were diluted to 350 or 100 nM concentration. In these conditions the microtubules spontaneously depolymerise so any effects of the kinesins upon this rate of depolymerisation should be easily detected. Klp5 and Klp6 were added to the microtubules in solution then aliquots removed and examined by darkfield microscopy. We found that the constructs Klp5436GST (B), Klp6440His (E) or Klp5436GST/Klp6440His (C) all caused bundling of the preformed microtubules under conditions where microtubules alone did not bundle. Removal of the GST tag from Klp5436 (D) or reduced ionic strength by omitting 100 mM KCl from the buffer or omitting ATP from buffer (data not shown) did not prevent bundling by this construct. Only Klp6415His, which omits the predicted dimerisation domain, did not cause any significant bundling even upon prolonged incubation (supplementary fig S5). These results suggest that the bundling activity, at least for Klp6 depends upon crosslinking via both of the kinesin heads in multiheaded constructs. It also supports Klp6 forming functional dimers or multimers. (TIF) [file pone.0030738.s009.tif]

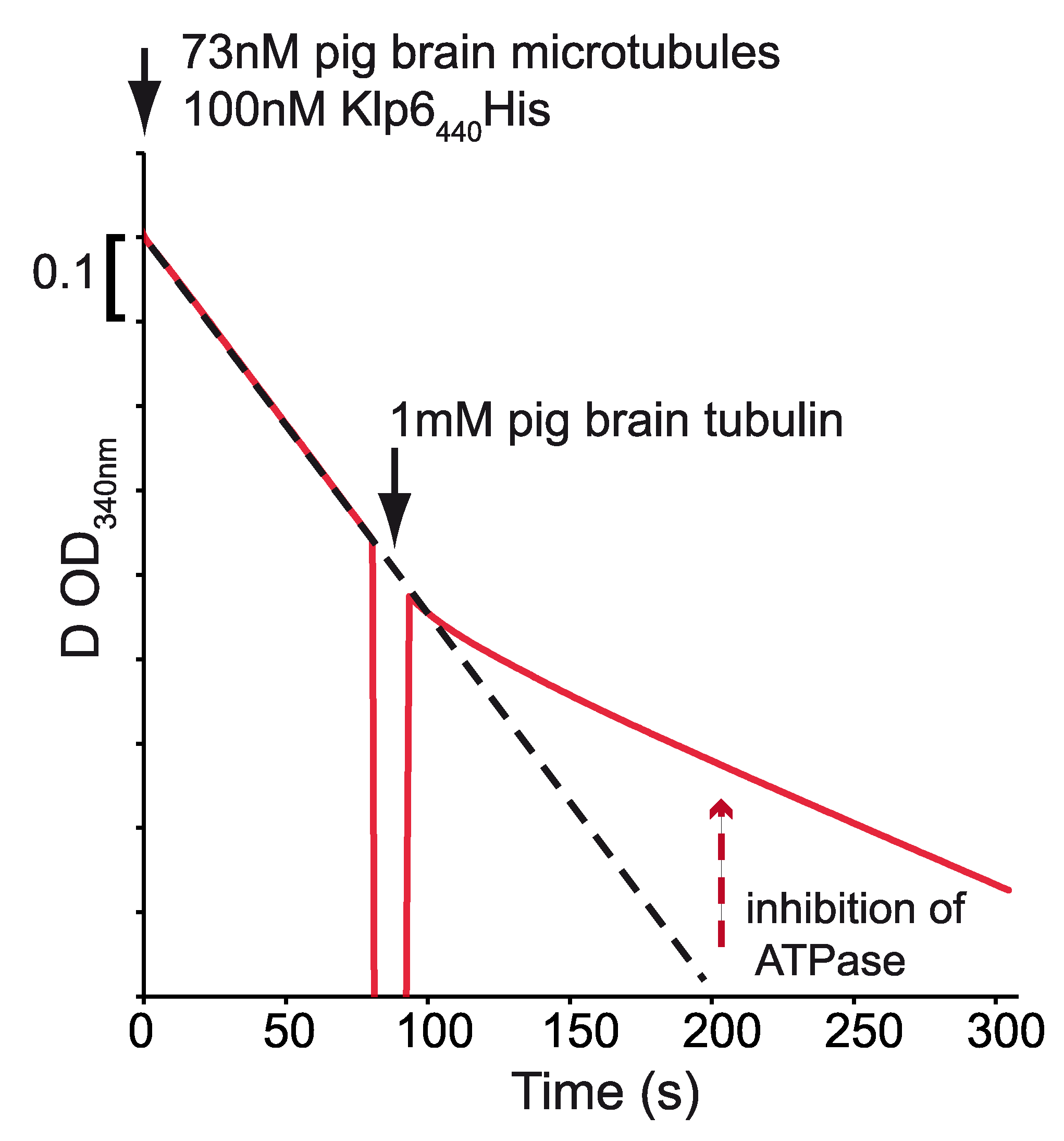

Supplement: Figure S10 — Klp6440His ATPase assay for competition between microtubule and tubulin heterodimer binding. 100 nM Klp6440His and 73 nM of Taxol stabilised pig brain microtubules were added to an ATPase assay where the decrease in absorbance at OD340 is directly linked to the ATPase activity in the assay. After incubation for 75 seconds pig brain tubulin heterodimers were added to 1 µM final concentration, with a break in monitoring during mixing. A new steady state of ATPase activity is then established rapidly. The decrease in slope of the new line (by 66% in the example shown) compared to the initial conditions (indicated by the dashed line) shows the inhibitory effect of the tubulin heterodimers in competition with the microtubules for activation of Klp440His ATPase. Typically the microtubule activated ATPase is inhibited within 20 sec following addition of an excess of pig brain tubulin and a new steady state of lower ATPase activity (due to the very low ATPase activation by the tubulin heterodimer) is established within ∼100 s. (TIF) [file pone.0030738.s010.tif]
