# Supplementary material for: S. pombe Kinesins-8 Promote Both Nucleation and Catastrophe of Microtubules
Source: PLoS One. 2012 Feb 20;7(2):e30738. doi: 10.1371/journal.pone.0030738 (PMC3282699; doi:10.1371/journal.pone.0030738)
Supplement: Table S1 — Microtubule Activated ATPase activity of full length Klp6FLHis. Microtubules were assembled from either pig brain tubulin and stabilised with Taxol or S. pombe tubulin stabilised with GMPCPP. Microtubule or tubulin heterodimer stimulated ATPase activities of Klp6FLHis were determined in linked assays at 25°C. Non-linear fits of plots of ATPase activity against tubulin concentration were used to determine Vmax and Km values. No tubulin stimulated ATPase activity was detected and Vmax and Km values for tubulin stimulation could not be determined. (DOC) [file pone.0030738.s017.doc]

**Table S1. Microtubule Activated ATPase activity of full length Klp6_FL_His.**

|  | **Microtubules** | | **Tubulin heterodimer** | |
| --- | --- | --- | --- | --- |
| **source** | **Vmax (s^-1^)** | **Km (nM)** | **Vmax (s^-1^)** | **Km (nM)** |
| **Pig brain** | 1.13 ± 0.07 | 16.3 ± 3.2 | None detected | NA |
| ***S. pombe*** | 0.96 ± 0.08 | 32.2 ± 9.1 | None detected | NA |

Vmax and Km values ± standard error of mean
