# Supplementary material for: S. pombe Kinesins-8 Promote Both Nucleation and Catastrophe of Microtubules
Source: PLoS One. 2012 Feb 20;7(2):e30738. doi: 10.1371/journal.pone.0030738 (PMC3282699; doi:10.1371/journal.pone.0030738)
Supplement: Table S2 — Microtubule Velocities reported for kinesins-8. Published values for microtubule sliding velocities on surfaces of Kinesin-8 motors and for velocity of single molecules of Kinesin-8 moving on microtubules. (DOC) [file pone.0030738.s018.doc]

**Table S2. Microtubule Velocities reported for kinesins-8.**

| **Kinesin-8** | **In vivo (nm s^-1^)** | **Gliding assay in vitro (nm s^-1^)** | **Single molecule in vitro (nm s^-1^)** |
| --- | --- | --- | --- |
| **Klp6MDN** |  | 56^(1), (i, ii, iii)^ |  |
| **Klp5/6 FL** |  | 9 ^(1), (i)^, 39 ^(1), (ii)^ |  |
| **Klp6 FL** |  | 2 ^(1), (i, ii)^ |  |
| **Kip3** | 74^(2)^ | 11^(2)^ | 50^(3)^ |
| **Kif18a** |  | 20^(4)^, 103^(5)^,126^(6), (iv)^, 174^(6), (v)^ |  |
| **Klp67A** |  | 50^(7)^ |  |

^i^ 0 mM NaCl added to assay

^ii^ 200mM NaCl added to assay

^iii^ Klp6MDN equivalent to our construct Klp6_440_His

^iv^ Kif18A-FL-GFP

^v^ Kif18A-N480-GFP

**References**

^1^ Grissom PM, Fiedler T, Grishchuk EL, Nicastro D, West RR, McIntosh JR (2009) Kinesin-8 from fission yeast: a heterodimeric, plus-end-directed motor that can couple microtubule depolymerization to cargo movement. *Mol Biol Cell* **20**: 963-972

^2^ Gupta Jr ML, Carvalho P, Roof DM, Pellman D (2006) Plus end-specific depolymerase activity of Kip3, a kinesin-8 protein, explains its role in positioning the yeast mitotic spindle. *Nature cell biology* **8**: 913-923

^3^ Varga V, Helenius J, Tanaka K, Hyman AA, Tanaka TU, Howard J (2006) Yeast kinesin-8 depolymerizes microtubules in a length-dependent manner. *Nature cell biology* **8**: 957-962

^4^ Mayr MI, Hummer S, Bormann J, Gruner T, Adio S, Woehlke G, Mayer TU (2007) The human kinesin Kif18A is a motile microtubule depolymerase essential for chromosome congression. *Curr Biol* **17**: 488-498

^5^ Du Y, English CA, Ohi R, (2010) The kinesin-8 Kif18A dampens microtubule plus-end dynamics. *Current Biology* **20**: 374-380

^6^ Su X, Qiu W, Gupta Jr ML, Pereira-Leal JB, Reck-Peterson, SL, Pellman D, (2011) Mechanisms underlying the dual-mode regulation of microtubule dynamics by kip3/kinesin-8*.* *Molecular cell* **43**: 751-763

^7^ Pereira AJ, Dalby B, Stewart RJ, Doxsey SJ, Goldstein LS (1997) Mitochondrial association of a plus end-directed microtubule motor expressed during mitosis in Drosophila. *J Cell Biol* 136: 1081-1090
