# Supplementary material for: S. pombe Kinesins-8 Promote Both Nucleation and Catastrophe of Microtubules
Source: PLoS One. 2012 Feb 20;7(2):e30738. doi: 10.1371/journal.pone.0030738 (PMC3282699; doi:10.1371/journal.pone.0030738)
Supplement: Table S3 — Velocity and dwell times of Klp6440His coated beads on pig brain tubulin microtubules. An optical trap was used to position Klp6440His coated beads on Taxol stabilised microtubules assembled from pig brain tubulin (figure 2). The velocity of bead translocation along the microtubule and subsequent dwell time at the microtubule tip was measured for 6 beads. The mean dwell time was 42±10 s (6) and velocity 47±7 nm/s (5) (mean ± SEM (n)). The average velocity is slower than is observed in motility assays (87±18 nm/s (1085)), which may be caused by different buffers or surface densities of Klp6440His in the two assays. Bead movement was processive at high motor concentrations. At low motor densities beads failed to attach and move along microtubules. (DOC) [file pone.0030738.s019.doc]

**Table S3. Velocity and dwell times of Klp6_440_His coated beads on pig brain tubulin microtubules.**

| **Bead** | **Bead velocity (nm s^-1^)** | **Dwell time (s)** |
| --- | --- | --- |
| **1** | 51.3 ± 8.5 (4) | 30 |
| **2** | 60.4 ± 4.4 (4) | 40 |
| **3** | ND | 33 |
| **4** | 46.0 ± 7.6 (5) | 11 |
| **5** | 57.5 ± 14.2 (6) | 80 |
| **6** | 20.5 ± 4.1 (9) | 57 |

mean ± SD (n)

ND - value not determined
