# Supplementary material for: S. pombe Kinesins-8 Promote Both Nucleation and Catastrophe of Microtubules
Source: PLoS One. 2012 Feb 20;7(2):e30738. doi: 10.1371/journal.pone.0030738 (PMC3282699; doi:10.1371/journal.pone.0030738)
Supplement: Table S4 — Effect of Klp436GST upon GMPCPP stabilised pig brain Microtubule depolymerisation rates. Microtubule bundling activity of Klp5 and klp6 (see supplementary figure S9) prevented detailed analysis of their effect upon microtubule stability in solution by microscopic methods, apart from noting that even in incubations lasting up to 70 min numerous bundled microtubules were still present at the end of the incubation period. To test if this bundling activity might be masking a depolymerase activity the effect of klp5436GST upon individual microtubules was tested by first binding single microtubules to a poly-lysine coated surface before addition of the kinesin and imaging by dark field microscopy. Time-lapse movies were recorded then analysed using the kymograph function of Metamorph software. Spontaneous depolymerisation of the GMPCPP microtubules at 0.40 nm s−1 was still observed. Although addition of Klp5436GST caused no bundling, depolymerisation was not significantly enhanced suggesting that bundling was not masking a depolymerase activity. (DOC) [file pone.0030738.s020.doc]

**Table S4. Effect of Klp_436_GST upon GMPCPP stabilised pig brain Microtubule depolymerisation rates.**

| **Klp5_436_GST (nM)** | **MT depolymerisation rate (nm s^-1^)** |
| --- | --- |
| 0 | 0.40 ± 0.05 (27) |
| 21 | 0.34 ± 0.03 (45) |
| 199 | 0.39 ± 0.05 (33) |
| 1461 | 0.51 ± 0.11 (16) |

mean ± SEM (n)
