# Supplementary material for: S. pombe Kinesins-8 Promote Both Nucleation and Catastrophe of Microtubules
Source: PLoS One. 2012 Feb 20;7(2):e30738. doi: 10.1371/journal.pone.0030738 (PMC3282699; doi:10.1371/journal.pone.0030738)
Supplement: Table S5 — His-Klp6FL effect upon S. pombe GTP microtubule fast end dynamics. Effect of His-Klp6FL on fast end microtubule dynamics in assays at 25°C containing 4.4 µM S. pombe GTP tubulin with microtubules nucleated by axoneme fragments. (DOC) [file pone.0030738.s021.doc]

**Table S5. His-Klp6_FL_ effect upon *S. pombe* GTP microtubule fast end dynamics.**

| **Klp6_FL_  (nM)** | **Growth (nm s^-1^)** | **Shrinkage (nm s^-1^)** | **Cat (min^-1^)** | **Res (min^-1^)** | **Growth (%)** | **Shrinkage (%)** | **Pause (%)** |
| --- | --- | --- | --- | --- | --- | --- | --- |
| **0** | 8.6 ± 1.1 (15) | 184 ± 23 (13) | 0.32 (11) | 1.32 (3) | 93.6 | 6.1 | 0.4 |
| **82** | 8.9 ± 0.6 (16) | 221 ± 23 (13) | 0.30 (11) | 0.53 (1) | 90.8 | 4.8 | 4.4 |
| **164** | 8.1 ± 0.8 (18) | 195 ± 18 (16) | 0.29 (12) | 0.80 (2) | 87.1 | 5.3 | 7.7 |

mean ± SEM (n)
