# Supplementary material for: S. pombe Kinesins-8 Promote Both Nucleation and Catastrophe of Microtubules
Source: PLoS One. 2012 Feb 20;7(2):e30738. doi: 10.1371/journal.pone.0030738 (PMC3282699; doi:10.1371/journal.pone.0030738)
Supplement: Table S6 — His-Klp6FL effect upon S. pombe GTP microtubule slow end dynamics. Effect of His-Klp6FL on slow end microtubule dynamics in assays at 25°C containing 4.4 µM S. pombe GTP tubulin with microtubules nucleated by axoneme fragments. (DOC) [file pone.0030738.s022.doc]

**Table S6. His-Klp6_FL_ effect upon *S. pombe* GTP microtubule slow end dynamics.**

| **Klp6_FL_ (nM)** | **Growth (nm s^-1^)** | **Shrinkage (nm s^-1^)** | **Cat (min^-1^)** | **Res (min^-1^)** | **Growth (%)** | **Shrinkage (%)** | **Pause (%)** |
| --- | --- | --- | --- | --- | --- | --- | --- |
| **0** | 6.1 ± 1.0 (9) | 80 (1) | 0.02 (1) | 6.0 (1) | 99.7 | 0.3 | 0 |
| **82** | 4.8 ± 0.2 (6) | none^2^ | 0^1^ | none^2^ | 100 | 0 | 0 |
| **164** | 4.6 ± 0.3 (8) | 100 ± 2 (2) | 0.05 (2) | 3.33 (1) | 99.3 | 0.7 | 0 |

mean ± SEM (n)

**^1^** 0 catastrophes in 4086 seconds growth

^2^no shrinkage and therefore no rescue events were observed.
