# Supplementary material for: S. pombe Kinesins-8 Promote Both Nucleation and Catastrophe of Microtubules
Source: PLoS One. 2012 Feb 20;7(2):e30738. doi: 10.1371/journal.pone.0030738 (PMC3282699; doi:10.1371/journal.pone.0030738)
Supplement: Table S7 — Klp5436GST/Klp6440His effect upon S. pombe GTP microtubule fast end dynamics. Effect of KLP5436GST/KLP6440His on fast end microtubule dynamics in assays at 25°C containing 4.0 µM S. pombe GTP tubulin with microtubules nucleated by axoneme fragments. (DOC) [file pone.0030738.s023.doc]

**Table S7. Klp5_436_GST/Klp6_440_His effect upon *S. pombe* GTP microtubule fast end dynamics.**

| **klp5/6 (nM)** | **Growth (nm s^-1^)** | **Shrinkage (nm s^-1^)** | **Cat (min^-1^)** | **Res (min^-1^)** | **Growth (%)** | **Shrinkage (%)** | **Pause (%)** |
| --- | --- | --- | --- | --- | --- | --- | --- |
| **0** | 8.5 ± 0.5 (15) | 192 ± 25 (14) | 0.19 (12) | 0.01 (4) | 92.3 | 6.5 | 1.2 |
| **372** | 7.9 ± 0.4 (16) | 233 ± 31 (12) | 0.18 (11) | 0^1^ | 94.7 | 5.1 | 1.2 |
| **42^3^** | 7.7 ± 0.4 (13) | 234 ± 24 (13) | 0.14 (9) | 0^2^ | 92.7 | 4.9 | 2.4 |

mean ± SEM (n)

^1^0 rescues in 197 sec of shrinkage

^2^0 rescues in 204 sec shrinkage

^3^MT affinity purified KLP5_436_GST/KLP6_440_His.
