# Supplementary material for: S. pombe Kinesins-8 Promote Both Nucleation and Catastrophe of Microtubules
Source: PLoS One. 2012 Feb 20;7(2):e30738. doi: 10.1371/journal.pone.0030738 (PMC3282699; doi:10.1371/journal.pone.0030738)
Supplement: Table S8 — Klp5436GST/Klp6440His effect upon S. pombe GTP microtubule slow end dynamics. Effect of KLP5436GST/KLP6440His on slow end microtubule dynamics in assays at 25°C containing 4.0 µM S. pombe GTP tubulin with microtubules nucleated by axoneme fragments. (DOC) [file pone.0030738.s024.doc]

**Table S8. Klp5_436_GST/Klp6_440_His effect upon *S. pombe* GTP microtubule slow end dynamics.**

| **klp5/6 (nM)** | **Growth (nm s^-1^)** | **Shrinkage (nm s^-1^)** | **Cat (min^-1^)** | **Res (min^-1^)** | **Growth (%)** | **Shrinkage (%)** | **Pause (%)** |
| --- | --- | --- | --- | --- | --- | --- | --- |
| **0** | 5.2 ± 0.3 (6) | none | 0^1^ | none | 100 | 0 | 0 |
| **372** | 5.5 ± 0.1 (6) | 175 ± 63 (2) | 0.05 (2) | 0^2^ | 82.0 | 1.8 | 16.2^4^ |
| **42^5^** | 5.2 ± 0.7 (9) | 618 ± 197 (2) | 0.04 (2) | 0^3^ | 99.6 | 0.4 | 0 |

mean ± SEM (n).

^1^0 catastrophes in 4826 sec of growth

^2^0 rescues in 56 sec shrinkage

^3^0 rescues in 12 sec shrinkage

^4^the increase in pausing is from a single MT

^5^MT affinity purified.
