# Supplementary material for: S. pombe Kinesins-8 Promote Both Nucleation and Catastrophe of Microtubules
Source: PLoS One. 2012 Feb 20;7(2):e30738. doi: 10.1371/journal.pone.0030738 (PMC3282699; doi:10.1371/journal.pone.0030738)
Supplement: Table S9 — Klp5436GST effect upon S. pombe GTP microtubule fast end dynamics. Effect of Klp5436GST on fast end microtubule dynamics in assays at 25°C containing 3.5 µM S. pombe GTP tubulin with microtubules nucleated by axoneme fragments. (DOC) [file pone.0030738.s025.doc]

**Table S9. Klp5_436_GST effect upon *S. pombe* GTP microtubule fast end dynamics.**

| **KLP5_436_GST (nM)** | **Growth (nm s^-1^)** | **Shrinkage (nm s^-1^)** | **Cat (min^-1^)** | **Res (min^-1^)** | **Growth (%)** | **Shrinkage (%)** | **Pause (%)** |
| --- | --- | --- | --- | --- | --- | --- | --- |
| **0** | 9.5 ± 0.7 (15) | 292 ± 24 (12) | 0.14 (11) | 0^1^ | 94.5 | 4.1 | 1.4 |
| **85** | 8.9 ± 1.2 (13) | 263 ± 17 (6) | 0.14 (6) | 0^2^ | 95.9 | 4.1 | 0 |
| **170** | 8.6 ± 0.1 (6) | 181 ± 50 (6)^5^ | 0.06 (3) | 0^3^ | 97.7 | 2.3 | 0 |
| **2960** | 5.1 ± 0.7 (4) | 31 ± 10 (5)^5^ | 0.22 (3) | 0.18 (2) | 38.4 | 31.8 | 29.8^4^ |

mean ± SEM (n)

^1^0 rescues in 206 sec shrinkage

^2^0 rescues in 112 sec shrinkage

^3^0 rescues in 70 sec of shrinkage

^4^2 MTs paused

^5^significantly different from 0 mM KLP5_436_GST rate p< 0.05
