# Supplementary material for: S. pombe Kinesins-8 Promote Both Nucleation and Catastrophe of Microtubules
Source: PLoS One. 2012 Feb 20;7(2):e30738. doi: 10.1371/journal.pone.0030738 (PMC3282699; doi:10.1371/journal.pone.0030738)
Supplement: Table S10 — Klp5436GST effect upon S. pombe GTP microtubule slow end dynamics. Effect of Klp5436GST on slow end microtubule dynamics in assays at 25°C containing 3.5 µM S. pombe GTP tubulin with microtubules nucleated by axoneme fragments. (DOC) [file pone.0030738.s026.doc]

**Table S10. Klp5_436_GST effect upon *S. pombe* GTP microtubule slow end dynamics.**

| **KLP5_436_GST (nM)** | **Growth (nm s^-1^)** | **Shrinkage (nm s^-1^)** | **Cat (min^-1^)** | **Res (min^-1^)** | **Growth (%)** | **Shrinkage (%)** | **Pause (%)** |
| --- | --- | --- | --- | --- | --- | --- | --- |
| **0** | 5.7 ± 0.4 (7) | none | 0^1^ | none | 95.8 | 0 | 4.2^4^ |
| **85** | 5.6 ± 0.3 (7) | none | 0^2^ | none | 100 | 0 | 0 |
| **170** | 5.3 ± 0.1 (6) | 78 ± 20 (2) | 0.03 (2) | 3.53 (2) | 99.1 | 0.9 | 0 |
| **2960** | 6.0 (1) | 66 (1) | 0^3^ | 1.88 (1) | 61.0 | 7.2 | 31.8^5^ |

mean ± SEM (n)

^1^0 catastrophe in 3966 sec growth

^2^0 catastrophe in 4362 sec growth

^3^0 catastrophe in 272 sec of growth

^4,5^1 pause event
