# Supplementary material for: S. pombe Kinesins-8 Promote Both Nucleation and Catastrophe of Microtubules
Source: PLoS One. 2012 Feb 20;7(2):e30738. doi: 10.1371/journal.pone.0030738 (PMC3282699; doi:10.1371/journal.pone.0030738)
Supplement: Table S11 — Oligonucleotides used in creating Klp5 and 6 constructs. Oligonucleotides used in PCR reactions to create the Klp5 and 6 protein expression vectors. (DOC) [file pone.0030738.s027.doc]

**Table S 11. Oligonucleotides used in creating Klp5 and 6 constructs**

| **Clone** | **Oligo name** | **Oligonucleotide sequence** |
| --- | --- | --- |
| **Klp6_440_** | Klp6f1 (BamH1site) | 5’ GTACAGGATCCCCTTTCTATGG 3’ |
|  | Klp6r2 (Stopcodon) | 5’CAGCTGCTCGAGTCAATGATGATGATGATGATGTCCTCCTTTTCGTACCTCCTTATTTAAAGC 3’ |
| **Klp6**_FL_ | Klp6r3 (FLstop) | 5’CAGCTGCTCGAGCCCGGGTCAATGATGATGATGATGATGGCCGCCAGCATTAGGAGTATTCTCAGT 3’ |
|  | Klp6f4 (N-His tag) | 5’CGCATATGGCAGGCCATCACCACCACCATCACGGCGCCATGAAAGAAGGGTCCTCTATTTC 3’ |
| **Klp5_436_** | Klp5f1 | 5’GATCGCATATGTCAAGACAGTCGTCCATTACCGTTACAGTCCGTGTTCGCCCATTTAGTAC 3’ |
|  | Klp5f2 | 5’GTCCGTGTTCGCCCATTTAGTACAG 3’ |
|  | Klp5f3 | 5’CTATCTTTGCCTATGGGGCAACTGG 3’ |
|  | Klp5r4 | 5’CCAGTTGCCCCATAGGCAAAGATAG 3’ |
|  | Klp5r5 | 5’GACGTCGAATTCCGGACCCTGAAACAGCACTTCCAGATTTGATTGCGATGACAAATC 3’ |
